# Supplementary material for: Integrating polygenic risk scores in the prediction of type 2 diabetes risk and subtypes in British Pakistanis and Bangladeshis: A population-based cohort study
Source: PLoS Med. 2022 May 19;19(5):e1003981. doi: 10.1371/journal.pmed.1003981 (PMC9119501; doi:10.1371/journal.pmed.1003981)
Supplement: S1 Text — (DOCX) [file pmed.1003981.s001.docx]

**S1 Text: Additional Methods**

*Quality control and imputation of genotype data in Genes & Health*

Standard quality control of samples (N=22,490) and variants was performed in Illumina’s GenomeStudio software. For duplicate samples and variants, the one with the highest call rate was retained. Variants with call rate <0.99 and minor allele frequency (MAF) <1% were removed using plink v1.9. Variants that failed the Hardy-Weinberg test (P-value <1✕10^-6^) in the 64% of the samples with low levels of autozygosity (i.e. proportion of the genome in runs of homozygosity <0.5%) were excluded.

To identify individuals of British Bangladeshi and British Pakistani (BPB) ancestry, we merged Genes & Health (G&H) with individuals of South Asian ancestry from the Human Genome Diversity Project (HGDP) [[1]](https://paperpile.com/c/r8E3bJ/RHqKK) and 1000 Genomes Project Phase 3 [[2]](https://paperpile.com/c/r8E3bJ/U5wit). We performed principal component analysis (PCA) in HGDP and 1000 Genomes samples, and projected G&H samples onto the same PC space using EIGENSOFT [[3]](https://paperpile.com/c/r8E3bJ/1PSiz). We then performed the uniform manifold approximation and projection dimension reduction method (UMAP) [[4]](https://paperpile.com/c/r8E3bJ/jdmuC) on the top 10 PCs using the R package “uwot”. We assigned British Pakistani and Bangladeshi ancestry to G&H samples that were in the same cluster with samples of Pakistani and Bangladeshi ancestry from external cohorts, respectively. We excluded individuals with reported ethnicity inconsistent with the genetically inferred ancestry.

The Michigan Imputation Server was used to perform imputation with the Genome Asia pilot reference panel. Palindromic single nucleotide polymorphisms (SNPs) and SNPs that were not found in the reference panel (including those with mismatched alleles) were excluded before imputation. We used different sets of imputed SNPs for different analyses in G&H:

- SNPs with INFO score ≥0.3 and MAF ≥0.1% (N=9,527,863) for constructing PRS
- SNPs with INFO ≥0.7 and MAF ≥0.5% (N=6,034,958) for genome-wide association analysis (GWAS)
- SNPs with INFO ≥0.9 and MAF ≥1% (N=3,118,071) for genetic correlation and colocalisation

For constructing a PRS, we find that not applying stringent QC (i.e. keeping more variants) is the best approach, especially when validating previously developed scores from the PGS Catalog. For genome wide association studies (GWAS), we used standard INFO and MAF filters. For trans-ancestry colocalisation analysis, which explicitly accounts for differences in linkage disequilibrium (LD) between ancestry populations, estimated directly from individual-level genotype data, we restricted the analysis to common variants with high imputation accuracy.

*Transferability of previously identified GWAS loci*

Previously identified loci associated with type 2 diabetes were obtained from the European-ancestry (EUR) GWAS (N= ~1.1 million) by Vujkovic *et al*. [[5]](https://paperpile.com/c/r8E3bJ/Biyo4). Unlike the construction of polygenic risk scores (PRS) for which the multi-ancestry GWAS data were used to increase power, we used European-ancestry GWAS to assess transferability of GWAS loci from European-ancestry into BPB individuals. The list of lead variants at each locus was from Supplementary Table 6 in the Vujkovic *et al* study. We performed GWAS of type 2 diabetes in G&H with SAIGE [[6]](https://paperpile.com/c/r8E3bJ/iIFUR) where sex, age, age^2^, and first 20 genetic PCs were adjusted for. We further excluded individuals with an EHR clinical code associated with being “at risk” of type 2 diabetes (**Table S1**), but not having developed the condition, leaving 12,785 individuals in GWAS. Following Huang *et al.* [[7]](https://paperpile.com/c/r8E3bJ/sTS8U), we assessed whether previously identified loci were reproducible in G&H at P-value <0.05. We addressed potential differences in LD between populations by considering all variants that were in high LD with the lead variant at each locus (i.e. the credible set), and assessed the transferability of all variants in credible sets. Credible sets for established loci consisted of lead (independent) variant and proxy SNPs (r^2^ >=0.8) within a 50kb window (based on the 1000 Genomes EUR data) of the sentinel variant and with p-value <100 ✕ p_sentinel_. This credible set is highly likely to contain the causal variant so we avoid a situation of a negative look up result due to population differences in LD. We assessed 338 loci from the Vujkovic *et al.* study that had variants in credible sets well-imputed in G&H. The expected power for replication was estimated assuming the same effect size as in the EUR discovery sample, and accounting for the allele frequency and sample size in G&H [[8,9]](https://paperpile.com/c/r8E3bJ/lhoF+YmP3). Pseudo-R code for calculating the power at each locus:

alpha = replication significance level

f = Allele frequency in controls in the replication cohort

n = sample size of the replication cohort

phi = n_case / n

b = effect size in the discovery GWAS

POWER = pchisq(qchisq(alpha, df = 1, lower = F), df = 1, ncp = 2*f*(1-f)*n*phi*(1-phi)*b^2, lower = F)

The number of loci that were expected to be transferable was estimated by summing up the power of lead variants across identified loci. The proportion of genetic loci one might expect to replicate was calculated by dividing the expected number of transferable loci over the number of all genetic loci identified in the European GWAS (n=338). We defined ‘transferable loci’ as those with at least one variant in the credible set associated with type 2 diabetes (P-value <0.05) in the same direction in G&H. We observed 76 (22.5%) transferable loci and the ratio of the number of observed transferable loci to that of expected (30.0%) was 0.75. 13 (3.8% out 338 loci tested) out of the 76 replicated loci were significant at P-value <1.5×10^-4^ (0.05/338; Bonferroni correction) in G&H, which was not significantly higher than the expectation estimated at alpha=0.05/338 (3.1%, one-sided binomial p-value 0.2). We defined ‘non-transferable’ loci as those with ≥1 variant in the credible set with >80% power but that had no variant in the credible set significant at P-value <0.05 and no variant located within 50kb from the locus significant at P-value <1✕10^-3^. We did not observe any loci that were well powered but not transferable in G&H.

We applied trans-ancestry colocalisation, which tests whether the association signals in two datasets can be explained by the same underlying causal variant without identifying the specific causal variant. Trans-ancestry colocalisation analysis was performed using TEColoc (<https://github.com/KarolineKuchenbaecker/TEColoc>) to assess whether a transferable locus shared the same causal variant between BPB and UK Biobank (UKBB) European (EUR) populations. TEColoc is described in detail by Kuchenbaecker *et al.* [[10]](https://paperpile.com/c/r8E3bJ/JD1yW). In brief, this method is based on the Joint Likelihood Mapping (JLIM) model developed by Chun *et al.* [[11]](https://paperpile.com/c/r8E3bJ/wl6bF). TEColoc takes individual-level genotype data and explicitly accounts for different LD structure between two populations, while more commonly used colocalisation methods, such as Giambartolomei *et al*. [[12]](https://paperpile.com/c/r8E3bJ/agt3h), assume that both summary statistics come from the same population so allele frequencies and patterns of LD are identical. For the reference sample set (the EUR GWAS), we calculated LD scores in the EUR superpopulation from the 1000 Genome Project phase 3. For BPB, we used genotype data to directly estimate LD for G&H. For each transferable locus, we used variants in a 50kb window that existed in both cohorts, and assessed loci with the proportion of overlapping SNPs ≥10% in both cohorts. The method assumes one causal variant in a given region, thus we used a small window of 50kb to minimise the possibility of including secondary causal signals. We could assess only 27 out of the 76 transferable loci, because we used common SNPs with high imputation quality (INFO ≥0.9 & MAF ≥1%) for the colocalisation analysis, and fewer well-imputed SNPs were available in G&H due to the much smaller imputation reference panel for South Asians than Europeans. We used a significance threshold of P-value <0.05 to determine evidence a causal variant was shared.

We applied the Popcorn algorithm to estimate the trans-ancestry genetic correlation between BPB and UKBB EUR populations using GWAS summary statistics [[13]](https://paperpile.com/c/r8E3bJ/DPqJO). LD scores were estimated using 1000 Genomes South Asian and EUR populations, excluding the major histocompatibility complex region.

*PRS using a South Asian-specific GWAS*

We assessed whether GWAS with matched ancestry could produce more accurate PRS. We first constructed a T2D PRS derived from the South Asian-specific GWAS by Kooner *et al.* [[14]](https://paperpile.com/c/r8E3bJ/aSBl), using the clumping and p-value thresholding method as described in Methods. This PRS did not show better performance (incremental area under curve (AUC) = 0.004 [CI 0.002–0.007]; OR = 1.15 [CI 1.10–1.20]) than the PRS reported in the main manuscript which was derived from the multi-ancestry GWAS by Vujkovic *et al*. [[5]](https://paperpile.com/c/r8E3bJ/Biyo4). This is expected given the small sample size of the South Asian GWAS, which is smaller than the participants from the Pakistani Genomic Resource included in the Vujkovic *et al*. study. Next, we attempted to combine the South Asian-specific PRS and the multi-ancestry PRS using the Marquez-Luna meta score approach [[15]](https://paperpile.com/c/r8E3bJ/2GSv). The two PRS were linearly combined using weights estimated from a logistic regression. The meta-PRS did not show improved accuracy (incremental AUC = 0.032 [CI 0.027–0.040]) compared to the muti-ancestry PRS alone. Thus, we used the muti-ancestry PRS in downstream analyses.

*PRS for insulin secretion and sensitivity measures*

We compared PRS for glycaemic traits between clusters. We first calculated PRS for beta-cell function developed by Udler *et al*. [*[16]*](https://paperpile.com/c/r8E3bJ/HfS1l). This beta-cell PRS was composed of 30 SNPs, and we used 29 of them that were well-imputed in G&H. We next calculated the type 2 diabetes weighted PRS developed by Mansour Aly *et al*. [[17]](https://paperpile.com/c/r8E3bJ/CIQQM). These scores were constructed using type 2 diabetes-associated SNPs weighted by their genetic effect on measures of insulin secretion and sensitivity, such as corrected insulin response (CIR). About 200 to 300 SNPs were used in calculating the weighted PRS.

To explore characteristics of the probable severe insulin deficient diabetes (pSIDD) cluster with previous reports of SIDD in the literature, mean PRS was compared between each cluster and non-diabetic controls (n = 10,841) using one-sided t tests (**Table S12**, **Fig S5A–B**). Next, following Mansour Aly *et al*. [[17]](https://paperpile.com/c/r8E3bJ/CIQQM), we calculated odds ratios of allocation of type 2 diabetes cases to each cluster compared to non-diabetic controls with increasing quintiles of each glycaemic trait PRS (**Fig 5C**).

*Power Calculation for Gestational Diabetes Analysis*

At the suggestion of a peer reviewer, We undertook an additional (retrospective) power analysis, taking an estimate in difference in means of 6.6% from Kwak *et al.* [[18]](https://paperpile.com/c/r8E3bJ/1NcYJ). At an alpha level of 0.05, the estimated required total sample size to identify a difference in means is n = 219, or n ~ 110 per group. Our sample size was n = 302 (with two groups of n = 175 and n = 127); therefore we expect this aspect of the analysis to be adequately powered.

**References**

1. [Li JZ, Absher DM, Tang H, Southwick AM, Casto AM, Ramachandran S, et al. Worldwide human relationships inferred from genome-wide patterns of variation. Science. 2008;319: 1100–1104.](http://paperpile.com/b/r8E3bJ/RHqKK)

2. [1000 Genomes Project Consortium, Auton A, Brooks LD, Durbin RM, Garrison EP, Kang HM, et al. A global reference for human genetic variation. Nature. 2015;526: 68–74.](http://paperpile.com/b/r8E3bJ/U5wit)

3. [Patterson N, Price AL, Reich D. Population structure and eigenanalysis. PLoS Genet. 2006;2: e190.](http://paperpile.com/b/r8E3bJ/1PSiz)

4. [McInnes L, Healy J, Melville J. UMAP: Uniform Manifold Approximation and Projection for Dimension Reduction. arXiv [stat.ML] [preprint]. 2018. Available:](http://paperpile.com/b/r8E3bJ/jdmuC) <http://arxiv.org/abs/1802.03426>

5. [Vujkovic M, Keaton JM, Lynch JA, Miller DR, Zhou J, Tcheandjieu C, et al. Discovery of 318 new risk loci for type 2 diabetes and related vascular outcomes among 1.4 million participants in a multi-ancestry meta-analysis. Nat Genet. 2020;52: 680–691.](http://paperpile.com/b/r8E3bJ/Biyo4)

6. [Zhou W, Nielsen JB, Fritsche LG, Dey R, Gabrielsen ME, Wolford BN, et al. Efficiently controlling for case-control imbalance and sample relatedness in large-scale genetic association studies. Nat Genet. 2018;50: 1335–1341.](http://paperpile.com/b/r8E3bJ/iIFUR)

7. [Huang QQ, Sallah N, Dunca D, Trivedi B, Hunt KA, Hodgson S, et al. Transferability of genetic loci and polygenic scores for cardiometabolic traits in British Pakistanis and Bangladeshis. medRxiv [preprint]. 2021; 2021.06.22.21259323.](http://paperpile.com/b/r8E3bJ/sTS8U)

8. [Sham PC, Purcell SM. Statistical power and significance testing in large-scale genetic studies. Nat Rev Genet. 2014;15: 335–346.](http://paperpile.com/b/r8E3bJ/lhoF)

9. [Matti Pirinen, University of Helsinki. GWAS 3: Statistical power. [cited 10 Feb 2022]. Available:](http://paperpile.com/b/r8E3bJ/YmP3) <https://www.mv.helsinki.fi/home/mjxpirin/GWAS_course/material/GWAS3.html>

10. [Kuchenbaecker K, Telkar N, Reiker T, Walters RG, Lin K, Eriksson A, et al. The transferability of lipid loci across African, Asian and European cohorts. Nat Commun. 2019;10: 4330.](http://paperpile.com/b/r8E3bJ/JD1yW)

11. [Chun S, Casparino A, Patsopoulos NA, Croteau-Chonka DC, Raby BA, De Jager PL, et al. Limited statistical evidence for shared genetic effects of eQTLs and autoimmune-disease-associated loci in three major immune-cell types. Nat Genet. 2017;49: 600–605.](http://paperpile.com/b/r8E3bJ/wl6bF)

12. [Giambartolomei C, Vukcevic D, Schadt EE, Franke L, Hingorani AD, Wallace C, et al. Bayesian test for colocalisation between pairs of genetic association studies using summary statistics. PLoS Genet. 2014;10: e1004383.](http://paperpile.com/b/r8E3bJ/agt3h)

13. [Brown BC, Asian Genetic Epidemiology Network Type 2 Diabetes Consortium, Ye CJ, Price AL, Zaitlen N. Transethnic Genetic-Correlation Estimates from Summary Statistics. Am J Hum Genet. 2016;99: 76.](http://paperpile.com/b/r8E3bJ/DPqJO)

14. [Kooner JS, Saleheen D, Sim X, Sehmi J, Zhang W, Frossard P, et al. Genome-wide association study in individuals of South Asian ancestry identifies six new type 2 diabetes susceptibility loci. Nat Genet. 2011;43: 984–989.](http://paperpile.com/b/r8E3bJ/aSBl)

15. [Márquez-Luna C, Loh P-R, South Asian Type 2 Diabetes (SAT2D) Consortium, SIGMA Type 2 Diabetes Consortium, Price AL. Multiethnic polygenic risk scores improve risk prediction in diverse populations. Genet Epidemiol. 2017;41: 811–823.](http://paperpile.com/b/r8E3bJ/2GSv)

16. [Udler MS, Kim J, von Grotthuss M, Bonàs-Guarch S, Cole JB, Chiou J, et al. Type 2 diabetes genetic loci informed by multi-trait associations point to disease mechanisms and subtypes: A soft clustering analysis. PLoS Med. 2018;15: e1002654.](http://paperpile.com/b/r8E3bJ/HfS1l)

17. [Mansour Aly D, Dwivedi OP, Prasad RB, Käräjämäki A, Hjort R, Thangam M, et al. Genome-wide association analyses highlight etiological differences underlying newly defined subtypes of diabetes. Nat Genet. 2021;53: 1534–1542.](http://paperpile.com/b/r8E3bJ/CIQQM)

18. [Kwak SH, Choi SH, Kim K, Jung HS, Cho YM, Lim S, et al. Prediction of type 2 diabetes in women with a history of gestational diabetes using a genetic risk score. Diabetologia. 2013;56: 2556–2563.](http://paperpile.com/b/r8E3bJ/1NcYJ)
