# Supplementary material for: Integrating polygenic risk scores in the prediction of type 2 diabetes risk and subtypes in British Pakistanis and Bangladeshis: A population-based cohort study
Source: PLoS Med. 2022 May 19;19(5):e1003981. doi: 10.1371/journal.pmed.1003981 (PMC9119501; doi:10.1371/journal.pmed.1003981)
Supplement: S1 Fig — AUC, area under the curve; GWAS, genome-wide association study; OR, odds ratio; PRS, polygenic risk score; SD, standard deviation. (DOCX) [file pmed.1003981.s003.docx]

**S1 Fig**: **Polygenic risk scores (PRSs) constructed using the clumping and P-value thresholding (C+T) method or from the PGS Catalog**.

**A**. Incremental area under the curve (AUC) of C+T PRSs across various P-value thresholds. The left panel shows the performance in the tuning set, which contained samples that were not included in the main analysis (2,249 cases and 4,443 controls; unrelated). Asterisk indicates the reported PRS with the highest predictive accuracy, which was used in subsequent analyses. The right panel shows the performance in samples used in the longitudinal analysis. The PRS that we reported is highlighted in the blue box.

**B**. Performance of the C+T PRS (red) and a previously developed score which showed the highest accuracy among all scores from the PGS Catalog (blue) in the samples used for longitudinal analysis (2,688 cases and 9,221 controls; unrelated). Incremental AUC and odds ratio (OR) per standard deviation (SD) of PRS are compared. Error bars represent 95% confidence intervals. The most accurate score from the PGS Catalog (PGS ID: PGS000020) was developed by Läll K *et al*. [[1]](https://paperpile.com/c/BWuNls/k7Dd9) using GWAS summary data from the DIAGRAM 2012 study [[2]](https://paperpile.com/c/BWuNls/3oVHO).


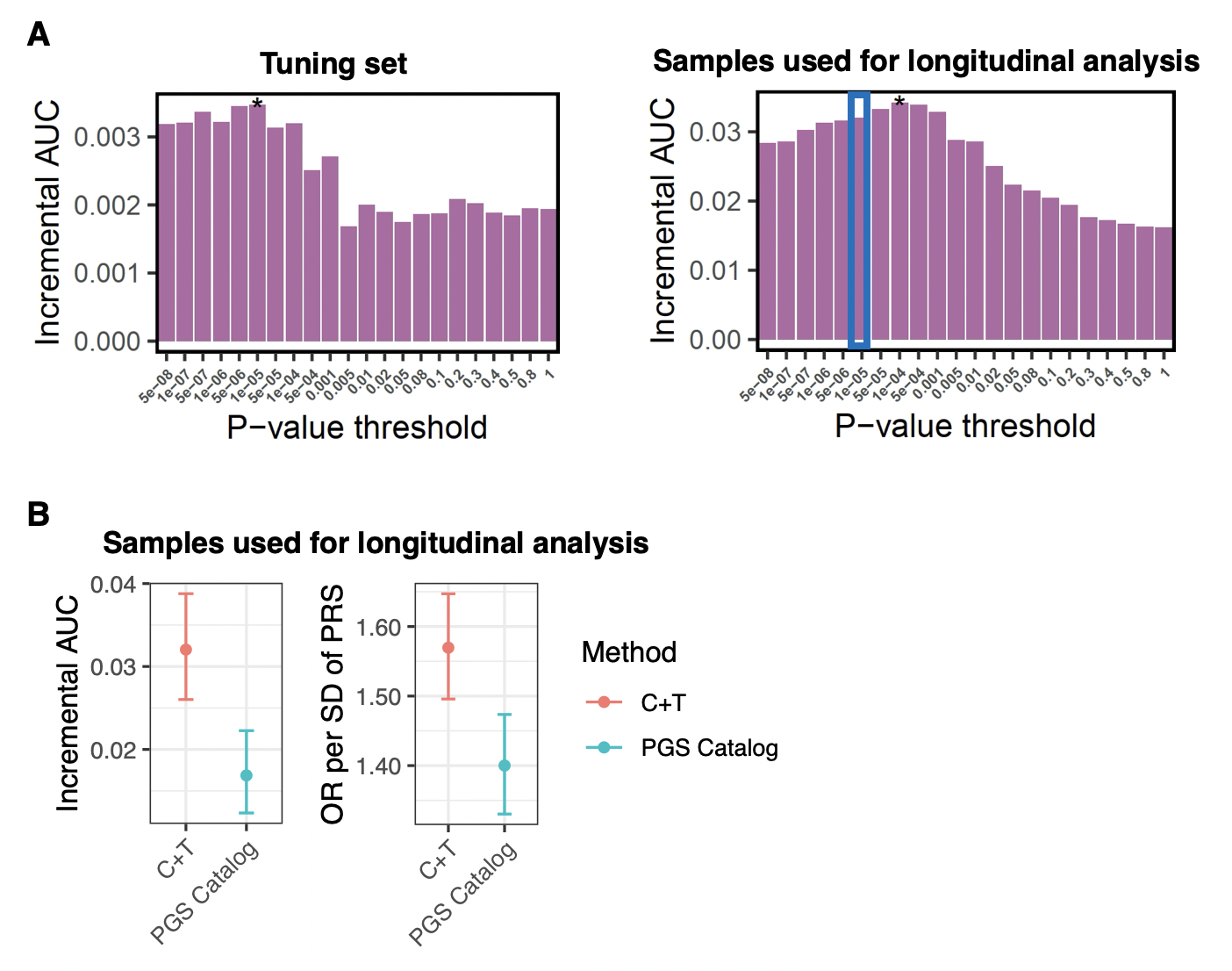


References

1. [Läll K, Mägi R, Morris A, Metspalu A, Fischer K. Personalized risk prediction for type 2 diabetes: the potential of genetic risk scores. Genet Med. 2017;19: 322–329.](http://paperpile.com/b/BWuNls/k7Dd9)

2. [Morris AP, Voight BF, Teslovich TM, Ferreira T, Segrè AV, Steinthorsdottir V, et al. Large-scale association analysis provides insights into the genetic architecture and pathophysiology of type 2 diabetes. Nat Genet. 2012;44: 981–990.](http://paperpile.com/b/BWuNls/3oVHO)
