# Supplementary material for: Integrating polygenic risk scores in the prediction of type 2 diabetes risk and subtypes in British Pakistanis and Bangladeshis: A population-based cohort study
Source: PLoS Med. 2022 May 19;19(5):e1003981. doi: 10.1371/journal.pmed.1003981 (PMC9119501; doi:10.1371/journal.pmed.1003981)

**S2 Fig:** **Effect of altering age cut-off for older and younger participants on net reclassification index in risk of incident type 2 diabetes analysis.**

Results are presented for QDiabetes Model A.


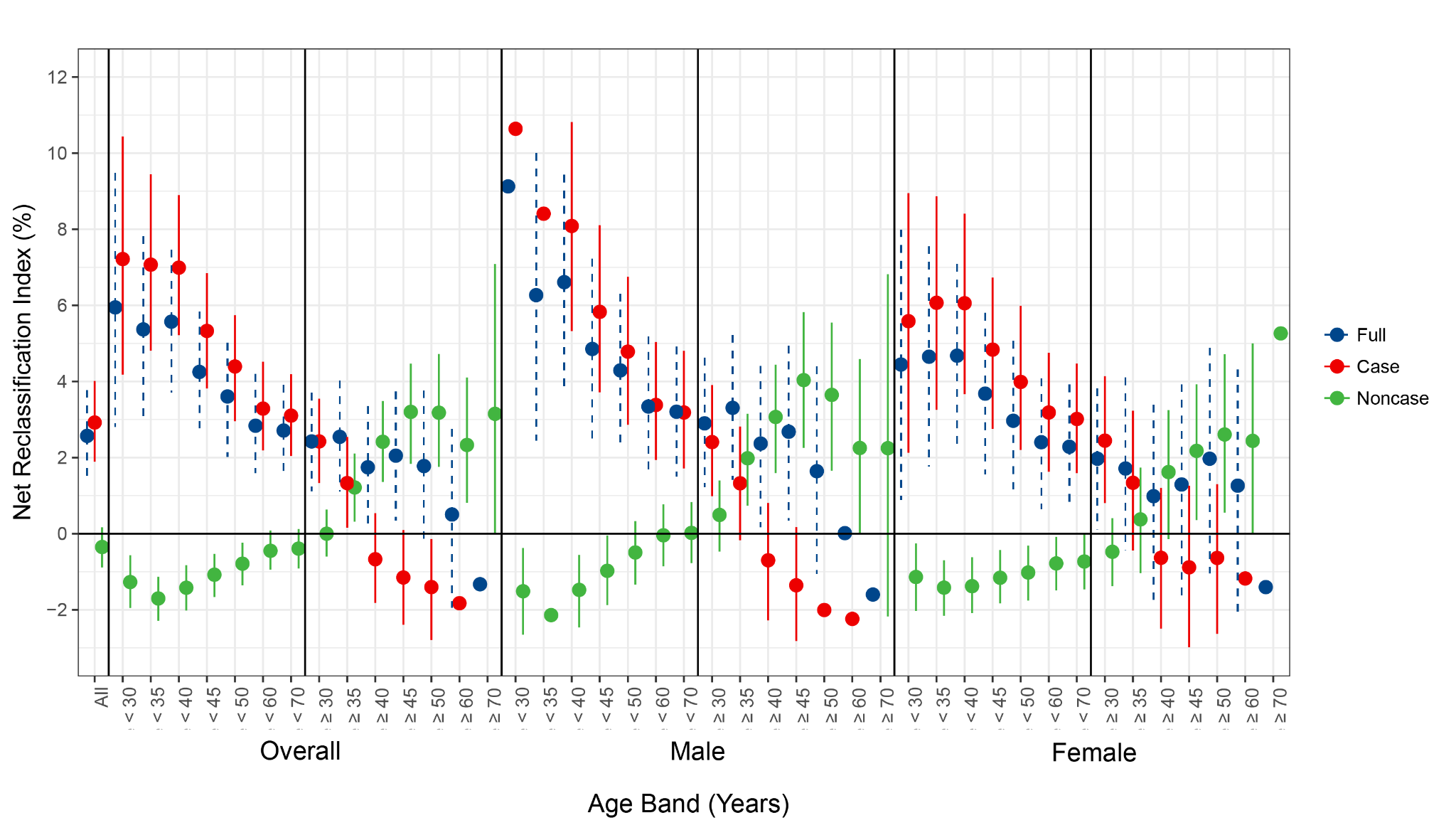

Supplement: S2 Fig — NRI, net reclassification index; T2D, type 2 diabetes. (DOCX) [file pmed.1003981.s004.docx]
