# Supplementary material for: Integrating polygenic risk scores in the prediction of type 2 diabetes risk and subtypes in British Pakistanis and Bangladeshis: A population-based cohort study
Source: PLoS Med. 2022 May 19;19(5):e1003981. doi: 10.1371/journal.pmed.1003981 (PMC9119501; doi:10.1371/journal.pmed.1003981)

**S3 Fig**: Elbow plot to identify optimal number of clusters in latent class analysis using Bayesian (BIC) and Akaike (AIC) information criteria.

AIC and BIC are penalised likelihood criteria which estimate the difference between the fitted likelihood function of a model, and the estimated true likelihood function of the data set. Lower values for AIC and BIC are associated with better model fit. For all model iterations the optimal number of clusters selected was 5.


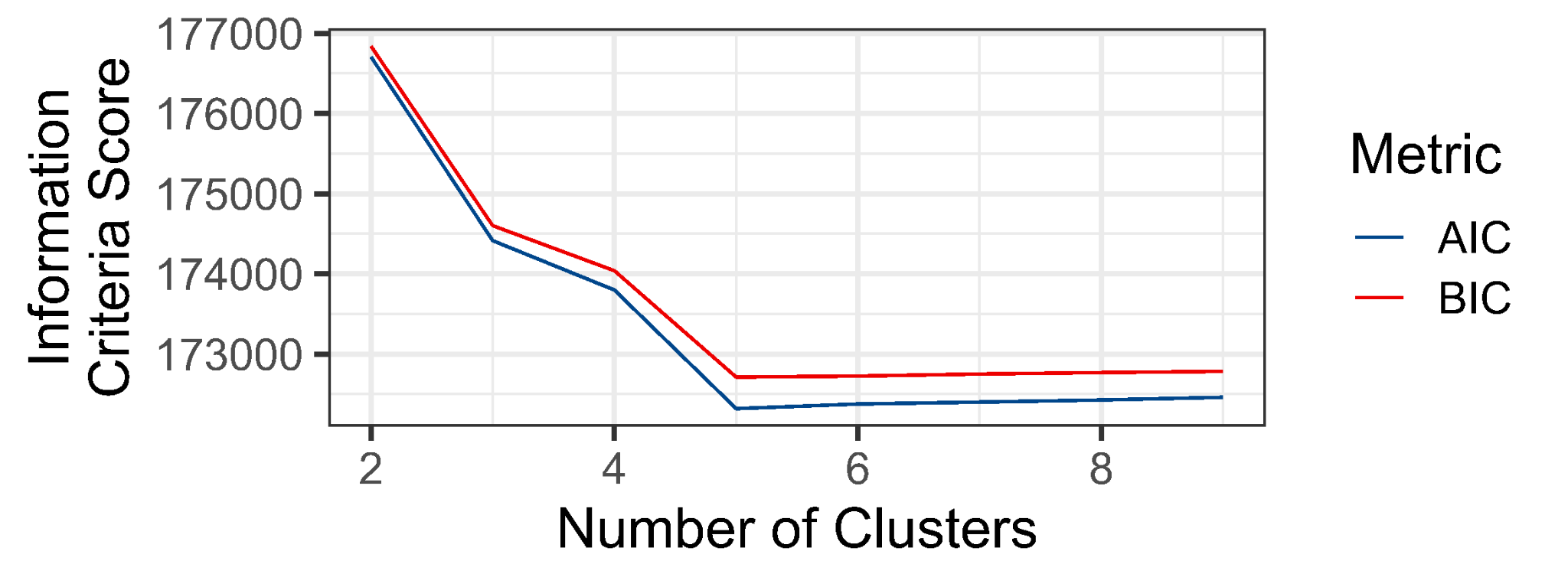

Supplement: S3 Fig — AIC, Akaike information criteria; BIC, Bayesian information criteria. (DOCX) [file pmed.1003981.s005.docx]
