# Supplementary material for: Integrating polygenic risk scores in the prediction of type 2 diabetes risk and subtypes in British Pakistanis and Bangladeshis: A population-based cohort study
Source: PLoS Med. 2022 May 19;19(5):e1003981. doi: 10.1371/journal.pmed.1003981 (PMC9119501; doi:10.1371/journal.pmed.1003981)

**S4 Fig**: Manhattan plot (**A**) and Q-Q plot (**B**) for the genome-wide association study (GWAS) in Genes & Health.

The lead SNPs at the three genome-wide significant (p-value < 5e-8) are in Table S3.


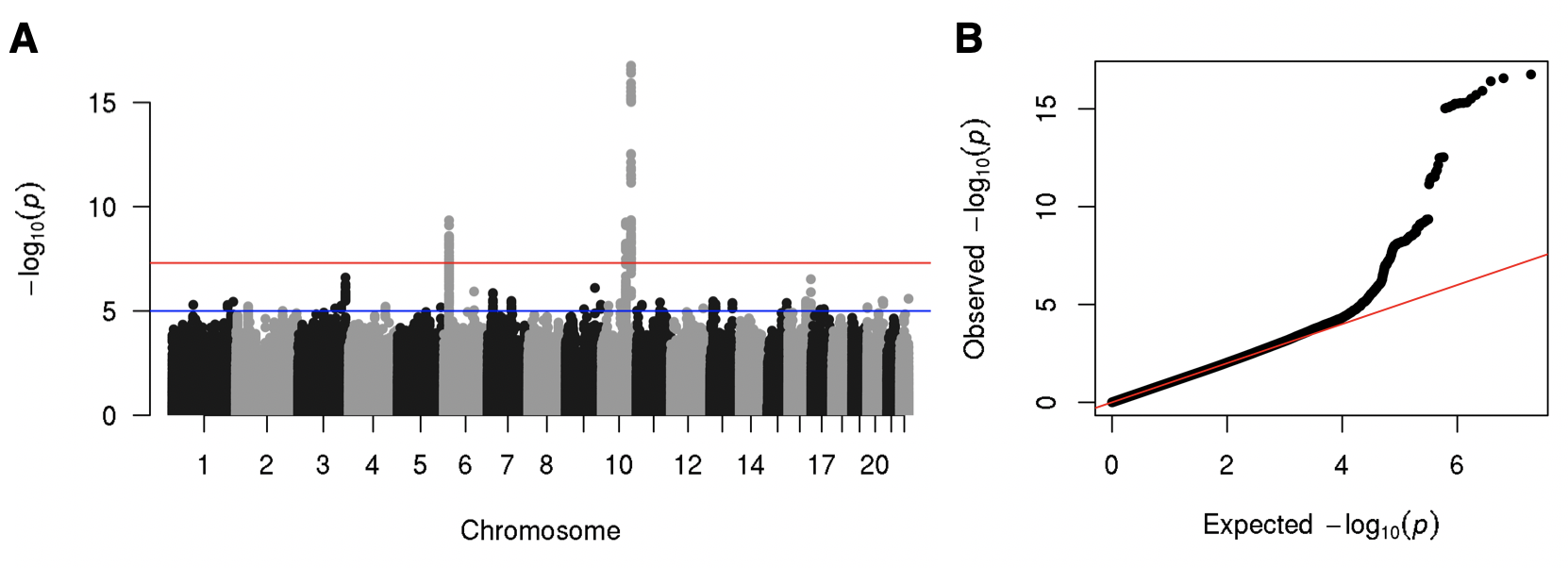

Supplement: S4 Fig — G&H, Genes & Health; GWAS, genome-wide association study. (DOCX) [file pmed.1003981.s006.docx]
