# Supplementary material for: Integrating polygenic risk scores in the prediction of type 2 diabetes risk and subtypes in British Pakistanis and Bangladeshis: A population-based cohort study
Source: PLoS Med. 2022 May 19;19(5):e1003981. doi: 10.1371/journal.pmed.1003981 (PMC9119501; doi:10.1371/journal.pmed.1003981)
Supplement: S5 Fig — BMI, body mass index; CI, confidence interval; CIR, corrected insulin response; FINS, fasting insulin; HbA1c, hemoglobin A1c; IRD, Insulin-Resistant Diabetes; ISI, insulin sensitivity index; MARD, Mild Age-Related Diabetes; MOD, Mild Obesity-related Diabetes; PRS, polygenic risk score; pSIDD, Probable Severe Insulin-Deficient Diabetes; SIRD, Severe Insulin-Resistant Diabetes; T2D, type 2 diabetes. (DOCX) [file pmed.1003981.s007.docx]

**S5 Fig**: Distribution of previously reported glycaemic trait polygenic risk scores (PRSs) across clusters in the Integrated Model, in which data-driven clusters were derived based on participant age, body mass index (BMI), HbA1c, serum triglycerides, and type 2 diabetes PRS.

In **Panel A**, the distribution of beta cell function PRS taken from Udler et al. [1] is compared between clusters.

In **Panel B**, the distribution of six glycaemic trait PRSs taken from Mansour Aly et al. [2] is compared between clusters. In **Panel C**, following Mansour Aly et al. [2], odds ratios of allocation to each of the five clusters in the Integrated Model compared to non-diabetic controls are presented by PRS quintile for 8 distinct PRSs. For each PRS, scores for all individuals included in analysis (n = 5,905 from Integrated Model) were determined and divided into quintiles. For each PRS, the odds ratio for allocation to each cluster compared to non-diabetic controls is presented alongside 95% confidence intervals (95%CI), with increasing PRS quintiles from left to right (in each case quintile 1 represents the lowest 20% of PRS scores and is used as the reference group, and quintile 5 the highest 20% of PRS scores). Across increasing type 2 diabetes PRS quintiles, for example, odds ratio for allocation to the pSIDD and IRD cluster increases, and for allocation to MARD and MOD decreases with increasing type 2 diabetes PRS quintiles, and decreases with increasing type 2 diabetes PRS. In Panel C, T2D PRS refers to the type 2 diabetes PRS constructed in Genes & Health, and Beta Cell refers to the PRS constructed by Udler et al [1] described in Panels A and B. The remaining 6 PRSs in Panel C represent weighted GRS (wGRS) scores for glycaemic traits as described by Mansour Aly et al. [2]. IRD - Insulin Resistant Diabetes; MARD - Mild Age Related Diabetes; MOD - Mild Obesity-Related Diabetes; pSIDD - probable Severe Insulin Deficient Diabetes; SIRD - Severe Insulin Resistant Diabetes; 2HGlucose - 2 hour Glucose wGRS; CIR - corrected insulin response (at 30 minutes during oral glucose tolerance test) wGRS; FINS - Fasting Insulin wGRS; ISI - insulin sensitivity index wGRS. Data are presented as mean with 95% confidence intervals.


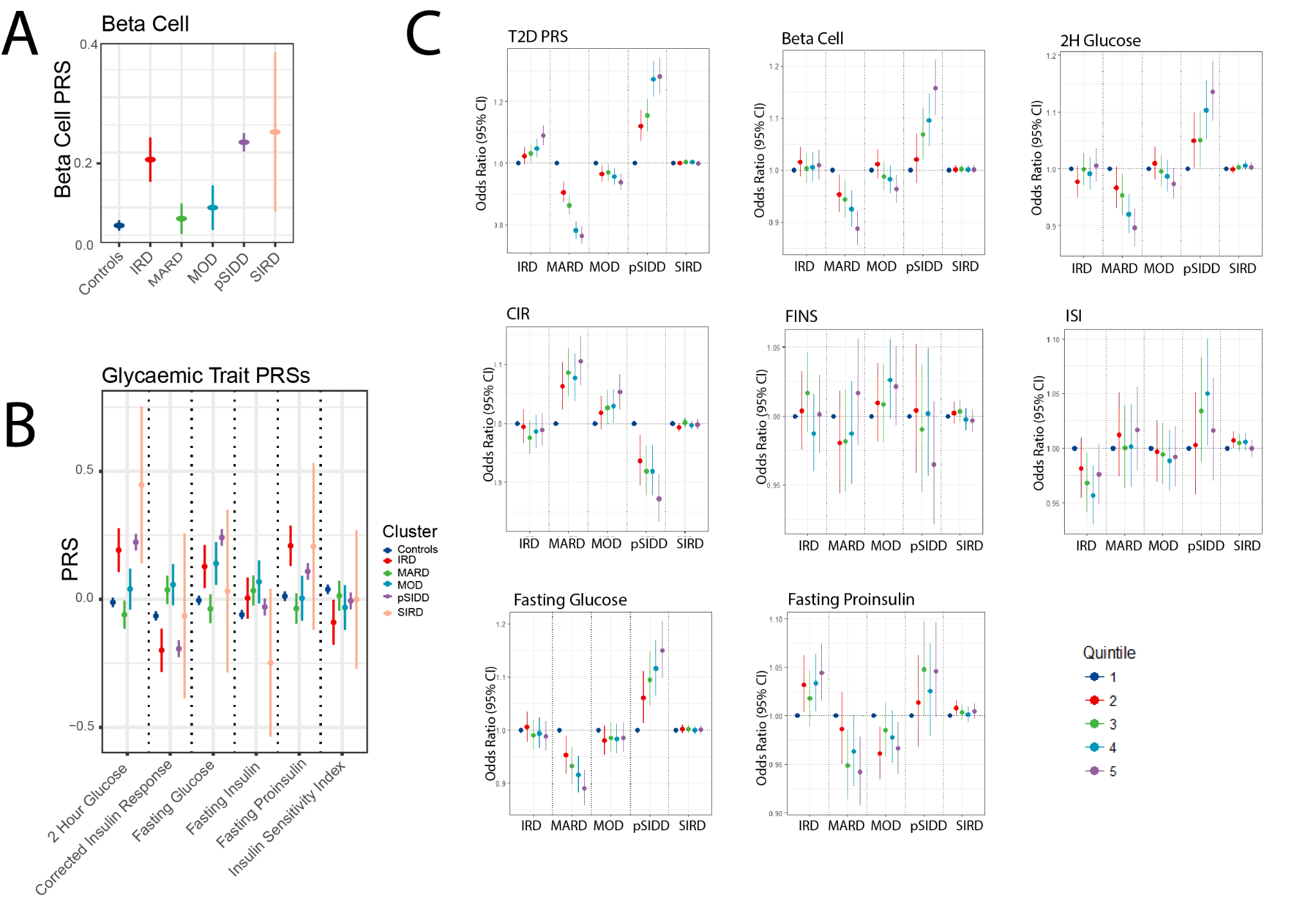


References

1. [Udler MS, Kim J, von Grotthuss M, Bonàs-Guarch S, Cole JB, Chiou J, et al. Type 2 diabetes genetic loci informed by multi-trait associations point to disease mechanisms and subtypes: A soft clustering analysis. PLoS Med. 2018;15: e1002654.](http://paperpile.com/b/BWuNls/TLXf)

2. [Mansour Aly D, Dwivedi OP, Prasad RB, Käräjämäki A, Hjort R, Thangam M, et al. Genome-wide association analyses highlight etiological differences underlying newly defined subtypes of diabetes. Nat Genet. 2021;53: 1534–1542.](http://paperpile.com/b/BWuNls/76Ti)
