# Supplementary material for: Integrating polygenic risk scores in the prediction of type 2 diabetes risk and subtypes in British Pakistanis and Bangladeshis: A population-based cohort study
Source: PLoS Med. 2022 May 19;19(5):e1003981. doi: 10.1371/journal.pmed.1003981 (PMC9119501; doi:10.1371/journal.pmed.1003981)

**S6 Fig**: Cox proportional hazard models to show association between cluster membership and development of macro- (**A**) and microvascular (**B**) complications of type 2 diabetes after adjustment for hypertension, statin use and serum cholesterol. Clusters are defined as Clinically Undifferentiated High Polygenic Susceptibility Diabetes (CUPS), Mild Age-Related Diabetes (MARD), Severe Insulin-Resistant Diabetes (SIRD), Insulin-Resistant Diabetes (IRD), and Mild Obesity-Related Diabetes (MOD).


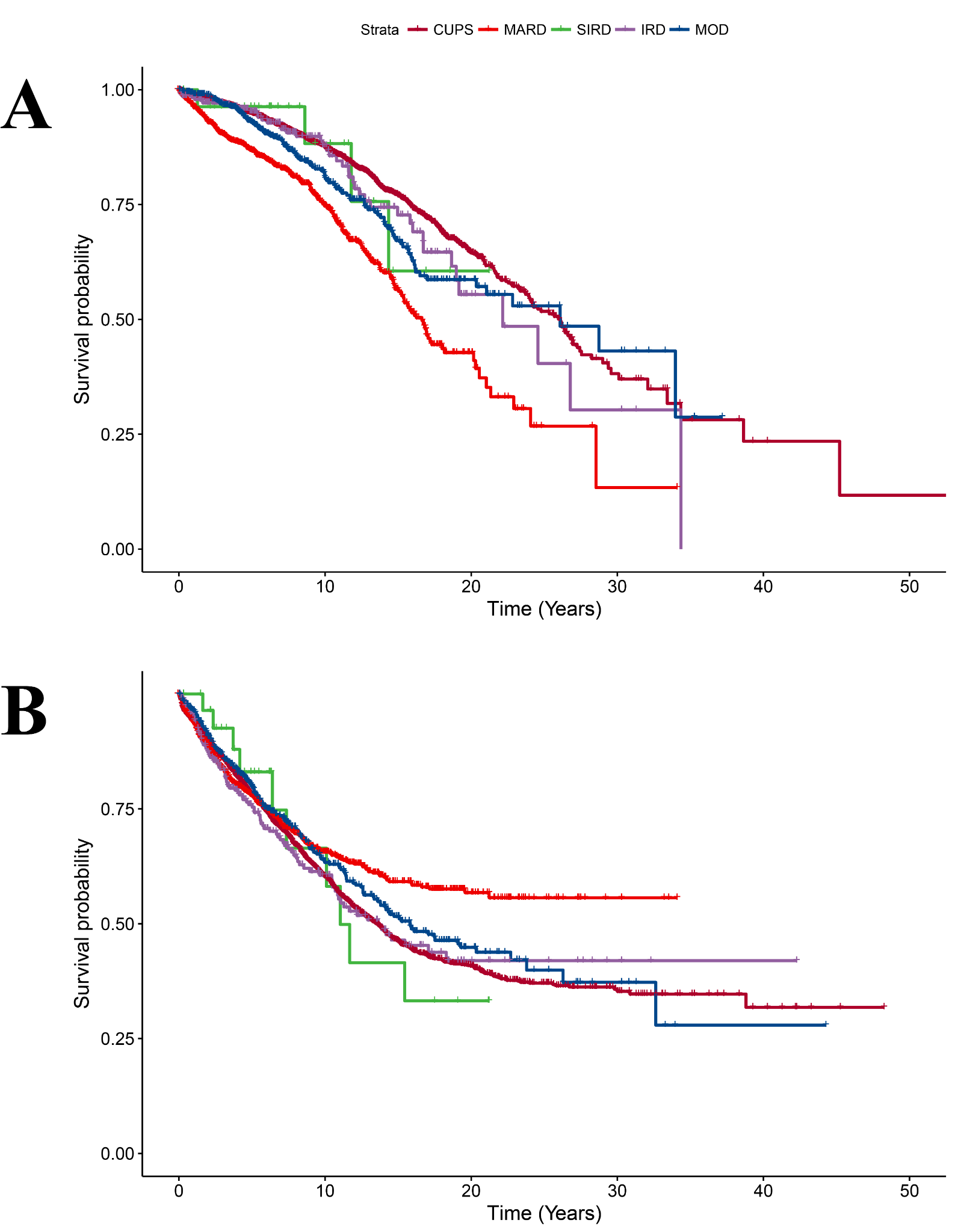

Supplement: S6 Fig — CUPS, Clinically Undifferentiated High Polygenic Susceptibility Diabetes; IRD, Insulin-Resistant Diabetes; MARD, Mild Age-Related Diabetes; MOD, Mild Obesity-related Diabetes; SIRD, Severe Insulin-Resistant Diabetes; T2D, type 2 diabetes. (DOCX) [file pmed.1003981.s008.docx]
