# Supplementary material for: Integrating polygenic risk scores in the prediction of type 2 diabetes risk and subtypes in British Pakistanis and Bangladeshis: A population-based cohort study
Source: PLoS Med. 2022 May 19;19(5):e1003981. doi: 10.1371/journal.pmed.1003981 (PMC9119501; doi:10.1371/journal.pmed.1003981)
Supplement: Attachment — Submitted filename: Response to Editors and Reviewers.docx [file pmed.1003981.s022.docx]

**Please see below for our detailed responses (in blue) to the Journal Editor, Academic Editor and Reviewers 1-3 (in black).**

**Journal Editor**

Title: Please revise your title according to PLOS Medicine's style. Your title must be nondeclarative and not a question. It should begin with main concept if possible. "Effect of" should be used only if causality can be inferred, i.e., for an RCT. Please place the study design ("A randomized controlled trial," "A retrospective study," "A modelling study," etc.) in the subtitle (ie, after a colon).

Thank you. We have changed this to “Integrating polygenic risk scores in the prediction of type 2 diabetes risk and subtypes: an analysis of genomic and routine primary care health data in a population-based study of British Pakistanis and Bangladeshis”

Abstract:

Abstract Background: Provide the context of why the study is important. The final sentence should clearly state the study question.
Abstract Methods and Findings:
* Please include the study design, years during which the study took place, and main outcome measures.
* Please quantify the main results (with 95% CIs and p values).
* Please include the important dependent variables that are adjusted for in the analyses.
* In the last sentence of the Abstract Methods and Findings section, please describe the main limitation(s) of the study's methodology.

Abstract Conclusions:
* Please address the study implications without overreaching what can be concluded from the data; the phrase "In this study, we observed ..." may be useful.
* Please avoid vague statements such as "these results have major implications for policy/clinical care". Mention only specific implications substantiated by the results.

Formatting:

Please add line numbering in the margins of the document to facilitate the review process.

Language:

Author summary bullet points 2 and 3: Please temper assertions of primacy (“No study has systematically…”) by adding ‘to our knowledge’ or similar. Please check throughout (other instances I noted were in the first and second paragraphs of the discussion). 

Please remove the final line of your introduction (“Our findings have major clinical relevance to a wider UK and global population of people of south Asian ancestry at risk of type 2 diabetes”). I would suggest toning this statement down and relocating it to the discussion.

Please remove funding information from the acknowledgements, in the event of publication this information is published as metadata based on your responses to the submission form questions.

For similar reasons, please also remove the conflict of interest and contributions statements from the end of the main text and ensure this information is captured as part of the submission form.

Please add ‘[preprint]’ after medRxiv for references 9, 13, and 41.

Please ensure you define all abbreviations used in your tables.

In Figures 1-3, please show the axis beginning at zero. If this is not possible, please show a break in the axis.

Thank you. We have made all of these changes.

Non-technical Author Summary:

At this stage, we ask that you include a short, non-technical Author Summary of your research to make findings accessible to a wide audience that includes both scientists and non-scientists. The Author Summary should immediately follow the Abstract in your revised manuscript. This text is subject to editorial change and should be distinct from the scientific abstract. Please see our author guidelines for more information: <https://journals.plos.org/plosmedicine/s/revising-your-manuscript#loc-author-summary>

Thank you. The Author Summary has now been edited considerably to meet these requirements.

Methods:

Did your study have a prospective protocol or analysis plan? Please state this (either way) early in the Methods section.

a) If a prospective analysis plan (from your funding proposal, IRB or other ethics committee submission, study protocol, or other planning document written before analyzing the data) was used in designing the study, please include the relevant prospectively written document with your revised manuscript as a Supporting Information file to be published alongside your study, and cite it in the Methods section. A legend for this file should be included at the end of your manuscript. 

b) If no such document exists, please make sure that the Methods section transparently describes when analyses were planned, and when/why any data-driven changes to analyses took place. 

c) In either case, changes in the analysis-- including those made in response to peer review comments-- should be identified as such in the Methods section of the paper, with rationale.

Please consider adding an additional figure providing an overview of the study design (see Figure 1 in <https://journals.plos.org/plosmedicine/article?id=10.1371/journal.pmed.1003498> for an example). This will aid comprehension for the non-specialist reader.

We did not create a formal prospective analysis plan before undertaking this study. We have amended the methods to clarify that “All analyses were planned at study outset, with the exception of the derivation of a second clustering model (Integrated Model), the rationale for which is given below” … “Having observed differing PRS distribution between clusters derived using clinical variables, we next explored whether inclusion of PRS as a clustering covariate influenced subgroup characteristics.”

We thank the reviewer for the suggestion of including an additional figure clarifying study design and analyses – we have included an additional figure (Figure 1) to this effect.

Reporting:

Please ensure that the study is reported according to the TRIPOD guideline, and include the completed TRIPOD checklist as Supporting Information. Please add the following statement, or similar, to the Methods: "This study is reported as per the Transparent Reporting of a Multivariable Prediction Model for Individual Prognosis or Diagnosis (TRIPOD) guideline (S1 Checklist)."

The TRIPOD guideline can be found here: <https://www.equator-network.org/reporting-guidelines/tripod-statement/>

When completing the checklist, please use section and paragraph numbers, rather than page numbers.

We have completed the checklist and added to the submission.

**Academic Editor**

Comment: 1. The diagnosis of diabetes was based on EMR (no OGTT was performed to screen for diabetes). This will have led to a low incidence of diabetes, and will have been largely affected by economic status.

We thank the editor for raising this point. Please also see the responses to Reviewer 2 who shared similar concerns.

While this is a clear limitation of the use of electronic medical records (and discussed as such in our strength and limitations section), this approach does mean findings are relevant to real world diagnosis and management of type 2 diabetes in primary care, particularly in the United Kingdom, where OGTT is no longer used (except in the diagnosis of gestational diabetes).

The population from which Genes & Health is sampled is socioeconomically deprived [[1]](https://paperpile.com/c/8EIZeG/Fu6P). However, whilst it might be hypothesised that this socioeconomic deprivation could lead to lower uptake of routine care and low incidence of diabetes, there is good evidence that this population have “health-seeking” behaviours that are associated with a high uptake of routine care, including type 2 diabetes screening via the NHS Health Check programme [[2]](https://paperpile.com/c/8EIZeG/KL28). We therefore anticipate that underdiagnosis rates in this population are similar, or lower, than national averages.

Comment 2. The genetic risk score was calculated based on the findings from European-ancestry individuals, I would suggest to use a South Asian genetic risk score or simply perform a replication for all reported loci for T2D with samples from South Asian individuals first.

We thank the editor for the suggestion. A reviewer had a similar suggestion - please also see the detailed responses to Reviewer 2, point 1.

We used the findings from, (a) European-ancestry individuals to assess the transferability of genetic loci from European-ancestry into South Asian-ancestry populations and, (b) used the more powerful multi-ancestry GWAS data to construct the T2D PRS. We apologise for the confusion and we have highlighted the different datasets used for different analyses in Methods (under the “Polygenic risk score construction”).

**Reviewer 1**

The authors perform a wide range of analysis to explore the genetic architecture of type 2 diabetes (T2D) in British Pakistanis and Bangladeshis (BPB). First the authors perform a genome-wide association study (GWAS) and contrast results with a large GWAS of European ancestry individuals. Secondly, the authors construct a polygenic risk score specific for BPB and check added predictive performance over the existing clinical score QDiabetes. My comments focus on the methodological details of these two main analysis steps.

1. Transfer of previously-identified GWAS loci to the study population

a) Please provide more details (e.g. citation, formulas or code) how the "expected power for replication" was calculated.

b) How did you calculate the expected proportion of genetic loci one might expect to replicate in a sample of a given ancestry given the power and linkage disequilibrium differences? Please provide more details (e.g. citation, formulas or code).

We thank the reviewer for raising these points. We have added the methodological details including pseudo-R code for calculating the expected power for replication in the Supplementary File 1 as the reviewer suggested. The number of genetic loci expected to replicate was calculated by first computing the power for each of the previously identified loci in G&H. This was based on the effect size from the discovery study and the sample size and allele frequency in G&H. By summing up the power of lead variants across identified loci, we derived the number we were powered to see replicate (expected number). The proportion was calculated by dividing the expected number over the number of all genetic loci identified in the European GWAS. In this analysis, we addressed potential differences in LD between populations by considering all variants that were in high LD with the lead variant at each locus (i.e. the credible set), and assessed the transferability of all variants in credible sets. This credible set is highly likely to contain the causal variant so we avoid a situation of a negative look-up result due to population differences in LD.

c) 76 loci are classified as "transferrable". Yet only 27 loci are analysed for colocalisation using TEColoc. Please explain why 49 loci are not considered. Is this due to the filter that ≥10% overlapping variants should be present? If so, please explain why there is so little overlap between Vujkovic et al European-ancestry GWAS and the BPB G&H GWAS.

We applied more stringent filtering (INFO >0.9 and MAF >1%) for the colocalisation analysis, which left about only half of the SNPs that were used in GWAS. We would also like to note that the imputation of South Asian populations is less accurate than European populations. We used a South Asian specific imputation panel (GenomeAsia pilot), which was much smaller than European reference panels, leading to fewer variants being well imputed in G&H. In addition, we used variants that existed in both cohorts, and assessed loci in which ≥10% of the variants from the GWAS were well imputed in G&H and vice versa. All these factors contributed to the small number of loci that were analysed in the colocalisation analysis. We have updated Supplementary File 1 accordingly.

d) Regarding imputation, in the Supplementary File 1 the number of SNPs used to construct PRS (N=9,527,863) is provided, but not the number for GWAS or colocalization analysis. Please provide these missing two numbers of SNPs as well. Why did you use different set of SNPs for different analysis?

We have now added the numbers of SNPs used in the other two analyses. When constructing a PRS, we find that *not* applying stringent QC (i.e. keeping more variants) is the best approach. For example, INFO 0.3 was used in [[3,4]](https://paperpile.com/c/8EIZeG/geV2+3H3o). For GWAS, we used INFO ≥0.7 and MAF ≥0.5% which are standard thresholds. For the trans-ancestry colocalisation analysis, which explicitly accounts for differences in LD between ancestry populations, applied directly to individual-level genotype data, we restricted the analysis to common variants with high imputation accuracy (INFO ≥0.9 and MAF ≥1%).

e) Please provide more details on the TEColoc or the JILM method and why you selected this method in contrast to the more commonly used colocalisation method by Giambartolomei et al.

We have added more details on the colocalisation method and the justification of our choice of the method in Supplementary File 1. In brief, the trans-ancestry colocalisation tests whether the association signals in two datasets can be explained by the same underlying causal variant without identifying the specific causal variant. The colocalisation method developed by Giambartolomei *et al.* [[5]](https://paperpile.com/c/8EIZeG/KQLj) (R “coloc” package [[6]](https://paperpile.com/c/8EIZeG/pIyt)) does not account for differences in LD, which is a major limitation in trans-ancestry colocalisation analysis. It assumes that both summary statistics come from the same ethnic population and allele frequencies and patterns of LD are identical. JLIM takes individual-level genotype data and accounts for different LD patterns between populations. For the reference sample set (the EUR GWAS), we calculated LD scores in the EUR superpopulation from the 1000 Genome Project phase 3. For BPB, we used genotype data to directly estimate LD for G&H.

2. Development of an integrated risk tool to predict incident type 2 diabetes

a) The authors show two ways to calculate the categorical net reclassification index (NRI): 
(1) the proportion of individuals who subsequently developed type 2 diabetes correctly reclassified as high risk, minus the proportion of individuals who subsequently developed type 2 diabetes incorrectly reclassified as low risk; 
(2) the proportion of individuals who did not develop type 2 diabetes correctly reclassified as low risk, minus the proportion of individuals who did not develop type 2 diabetes incorrectly reclassified as high risk.
Please be more precise how the proportions are defined, what exactly is the numerator and the denominator of the proportion?

b) Which of the two definitions was used for Figure 1 (Figure 2 in the revised manuscript) and in the Results?

Thank you for the opportunity to improve our previously unclear wording on the testing and performance of our integrated risk tool. For net reclassification index, we used the “full” proportion obtained by summing together definitions (1) and (2), ie, NRI = (1) + (2). The equation we have added (line 39, page 5) for clarity is NRI = (P(up|case) - P(down|case)) + (P(down|noncase) - P(up|noncase)). We have also now given detailed, but more salient, information regarding the denominator and numerator populations in a new Figure, Figure 1.

c) Figure 1 (Figure 2 in the revised manuscript) shows the NRI for all subjects and cases and non-cases. How can the "proportion of individuals who subsequently developed type 2 diabetes" be calculated for non-cases?

For non-cases, we calculated definition (2), which is the proportion of non-cases who were reclassified as low-risk individuals minus the proportion of non-cases who were reclassified as high-risk individuals.

d) There is a big distinction in the pattern of NRI for subjects younger or older than 40. How robust are the results with respect to this cut-off?

To explore the robustness of our results with respect to an age cut-off of 40 years between younger and older participants, we performed an additional analysis assessing the effect of varying age cut-off on net reclassification index (Additional supplementary figure S4 added). For example, for model A, re-running analysis with varying age cutoffs from 30-70, we saw a similar trend of NRI patterns as we did with the age cutoff of 40 up to age 60, beyond which the 95% confidence interval for the NRI begin to cross 0 for males, females and all participants above the age cutoff (but not those below the age cutoff). Prediction remains better in those below the age cutoff than those above the age cutoff for all age cutoffs tested up to 70 years. These results are presented in an additionally included figure (S4), and the methods updated (line 47, page 5) to include reference to this additional analysis suggested at the peer review stage. We additionally provide a further reference, with added clarity in the methods (line 48-9, page 5), which reflects an increasing body of work on early vs. late onset diabetes in which age 40 is used as a cutoff.

Minor
- Page 5: "with descriptive statistics. ." Please remove the second full period.

Thank you for catching this typo. This has been fixed.

**Reviewer 2**

Comment: In this paper, Hodgson et al. construct polygenic risk scores and integrate them with clinical models to enhance the prediction of type 2 diabetes 
 (T2D) in UK Pakistani and Bangladeshi individuals in a large population study. They first assess the transferability of known T2D genetic risk loci (derived from predominantly European populations) to their cohort and replicated significantly fewer loci than predicted. They then constructed a number of polygenic scores using variants identified from a multi-ancestry GWAS and adjusted/tuned the scores for Pakistani and Bangladeshi individuals in 2249 cases and 4443 controls, selecting the one with the highest odds for prediction. This score was then integrated with three versions of a clinical model (QDiabetes) to predict onset of T2D over 10 years in 13,642 individuals free from diabetes at baseline.  The authors show that incorporation of the PRS with the QDiabetes provided better discrimination of risk of progression to T2D, especially in those developing T2D <40 years. The PRS also was noted to be higher in women with a history of gestational diabetes who progressed to T2D versus non-progressors. Finally, they incorporated the PRS into a cluster analysis of baseline routine clinical characteristics, first largely replicating clusters defined previously in white populations, but also defining a 'clinically undifferentiated' cluster, associated with higher PRS but with no relationship to risk of progression to microvascular and macrovascular complications.

This study has several strengths:
 
1.      The analysis was conducted in a large population-based cohort of a hitherto understudied ethnic group; UK Pakistani and Bangladeshi individuals, who are at a disproportionately higher risk of developing T2D even within the south Asian ethnic group and at relatively lean body mass. It would be reasonable to postulate that this underlying risk is, in part, mediated by genetic predisposition.

2.      Longitudinal data linkage of participants to their electronic health record further provides a very powerful methodology in which genetic data can be associated with clinical outcomes. There are very few longitudinal cohorts of this size with linked data.

3.      This study uses multimodal approaches to decipher phenotype-genotype associations, including cluster analyses to identify heterogeneity in T2D sub-phenotypes, which is relatively novel in this ethnic group.

We thank the reviewer for highlighting these strengths of our paper.

On the other hand, there are some issues that must be addressed to enhance the relevance and focus of the paper:

1. The authors chose to apply polygenic scores derived in predominantly white European ethnicity to their Pakistani and Bangladeshi population and 'fine tuned' the effect size of variants to enable the best prediction of T2D. If their only goal had been to examine the transferrability of European PRS to South Asians, this may have been justified; however, their stated goal in the first sentence of the Discussion (also the more clinically relevant one) is to improve the prediction of T2D in South Asians via genetic means, and in that case the genetic predictive tool should include variants discovered in this population. One alternative, somewhat hindered by sample size, would have been to leverage the GWAS performed on cases and controls in this specific G&H cohort, and incorporate those variants into the score. Arguably, higher rates of consanguinity in this ethnicity have the potential to uncover novel loci. Nevertheless, at a minimum, the variants discovered by Kooner et al. (Nature Genetics 2011) should have also been included, as the trans-ancestry GWAS used to identify T2D loci has a very small number of South Asian individuals. The decision not to include South Asian-specific variants should be discussed or justified.

To clarify, we used, (a) a European-specific GWAS to assess the transferability of genetic loci, and, (b) the more powerful multi-ancestry GWAS (which contained South Asian individuals) to construct the PRS. We apologise for the confusing wording of our original manuscript. We have now highlighted the different datasets used for different analyses in Methods (under the “Polygenic risk score construction”).

We thank the reviewer for the suggestion of using South Asian-specific GWAS to build a PRS. We did not use Genes & Health to do this as conducting a GWAS and performing PRS validation in the same cohort would overestimate the performance of the PRS, and the cohort was not big enough to subdivide. Thus, in response to this reviewer’s comment, we explored several other approaches.

1. We tried constructing a T2D PRS using the South Asian-specific GWAS by Kooner *et al.* [[7]](https://paperpile.com/c/8EIZeG/Z1vh), which had a similar sample size with G&H. However, due to its small sample size, as pointed out by the reviewer, this South Asian PRS did not show better performance (incremental AUC = 0.004 [CI 0.002–0.007]; OR = 1.15 [CI 1.10–1.20]) than the PRS derived from the multi-ancestry GWAS by Vujkovic *et al.* [[8]](https://paperpile.com/c/8EIZeG/7M0H) that we reported in the paper (incremental AUC = 0.032 [CI 0.026–0.039]; OR = 1.57 [CI 1.50–1.65]).
2. We also attempted to combine the two PRSs using the Marquez-Luna metaGRS method [[9]](https://paperpile.com/c/8EIZeG/By10). However, this approach did not show improved accuracy either (incremental AUC = 0.032 [CI 0.027–0.040]).

The multi-ancestry GWAS by Vujkovic *et al.* contained 9.0k cases and 13.1k controls from the Pakistani Genomic Resource. The sample size for the Pakistanis is actually larger than the discovery cohort in the Kooner *et al.* 2011 study (5.6k cases and 14.5k controls). Thus it is not surprising that the large muti-ancestry GWAS conducted in 1.4 million samples from diverse ancestry led to a much more powerful PRS, and for this reason we are confident that this is the correct one to have used.

We have not integrated the results from these extra analyses into the paper but would be happy to do so should the reviewer or editor think it would be helpful.

2. In this vein, the GWAS conducted in G&H should be at least briefly presented, including the standard Manhattan and Q-Q plots and a table of the genome-wide significant associations, and placed in the context of the prior Kooner et al. GWAS.

We thank the reviewer for the suggestion. We have added the Manhattan and Q-Q plots in Figure S3. We identified 3 genome-wide significant loci (Table S4). None of them reached genome-wide significance in the Kooner *et al.* 2011 study [[7]](https://paperpile.com/c/8EIZeG/Z1vh), but all of them were identified in the larger multi-ancestry GWAS by Vujkovic *et al*. [[8]](https://paperpile.com/c/8EIZeG/7M0H). Among the 6 genetic loci reported by Kooner *et al.*, 4 were replicated in G&H at p-value < 0.05 (Table S5). We added this result in the second paragraph of the Results section.

3. One other major limitation is the absence of C-peptide or fasting insulin in this cohort, which pre-empts the ability to generate clusters analogous to those described by Ahlqvist et al. (ref. 19). Since this is an essential marker to denote beta-cell function, the authors are unable to reconstruct the "severe insulin deficient diabetes" (SIDD) cluster originally reported by Ahlqvist et al. Indeed, it is curious that what they term a novel "clinically undifferentiated" cluster has the strongest association with the T2D PRS. This is very telling: a close look at Supplementary Table 4 (Table S7 in the revised manuscript) reveals that the PRS constructed here is largely composed of the variants that have the strongest effect sizes on T2D (i.e. those that were originally discovered in Europeans but replicate in the current smaller sample). Therefore they are the ones that emerged from the earliest European GWAS, which by design favored loci that caused defects in beta-cell function (e.g. TCF7L2, MTNR1B, SLC30A8, KCNJ11, HNF4A, CDKAL1, IGF2BP2; see Pubmed ID 18504548 for an in-depth discussion of how the early T2D GWAS yielded beta-cell genes). Thus it is quite possible that the "clinically undifferentiated" cluster might indeed correspond to a cluster of severe, genetically driven beta-cell dysfunction, analogous to the SIDD cluster of Ahlqvist et al. As it turns out, a genetic exploration of the Ahlqvist clusters already available in preprint form (<https://www.medrxiv.org/content/10.1101/2020.09.29.20203935v1>) does demonstrate the strongest association of the T2D PRS with the SIDD cluster.

Thank you. Indeed the lack of C-peptide and fasting insulin data is a limitation, however it does also allow us to explore the potential clinical utility of PRSs in routine primary care data where these measures are not commonly available.

We very much appreciate the reviewer’s expertise and thoughts on our clusters analyses and interpretation. As noted by the reviewer, the high PRS associated with the “clinically undifferentiated” cluster indicates aetiology from beta-cell dysfunction, due to the representation of beta-cell genes in the variants included in the underlying GWAS and the build of our PRS. In addition, the low BMI and young age of people in this cluster, compared to the other clusters, is supportive of insulin deficiency. This is also supported by the recent publication of new clustering papers, including Prasad et al. [[10]](https://paperpile.com/c/8EIZeG/IfOO) which clearly identifies at SIDD cluster in another south Asian cohort as well as the recent paper by Mansour Aly et al. which combines clinical variables and PRS and which also identifies a strong association between SIDD and PRS in cluster analyses [[11]](https://paperpile.com/c/8EIZeG/4Voh). We therefore have renamed this cluster Probable Severe Insulin Deficient Diabetes (pSIDD), and expanded the interpretation of the relevant results and discussion. To further justify this definition, we have explored additional polygenic risk scores denoting additional diabetes-related traits replicated from the literature to examine the mean differences between the pSIDD cluster and non-diabetic controls (Figure S5, Table S12, updated results section). Mean fasting glucose and beta cell PRS were significantly higher in pSIDD than non-diabetic controls, in keeping with previous reports in the literature [[11,13]](https://paperpile.com/c/8EIZeG/7yHA+4Voh). In addition, we find increasing odds of allocation to pSIDD with increasing quintiles of fasting glucose PRS and beta cell PRS, and decreasing odds of allocation to pSIDD with increasing corrected insulin response PRS, in keeping with the extensive characterisation of the SIDD cluster by the Ahlquvist group [[11,13]](https://paperpile.com/c/8EIZeG/7yHA+4Voh).

4. The authors do not discuss a clear limitation that the time to follow-up of 10 years does not conclusively distinguish cases from controls, in that some participants classified as controls will eventually develop diabetes. Therefore, scores in the control group, given the high rates of T2D in this population are likely to be higher than true diabetes-free controls. Indeed, the mean age of participants in the dataset used to construct scores was 30.8 years (supp Table 2). Could they not have chosen 'older' diabetes free controls? Further the controls used to construct polygenic scores are described as having 'insufficient data to be included in the study' - it is not clear what is meant by this.

We thank the reviewer for highlighting these valuable points. In response to the reviewer’s first point (that some controls may in fact be cases), we have updated our strengths and limitations to highlight this. However we would expect this to mean that PRS scores may be slightly higher in our control group than true controls, therefore leading to an attenuation of our findings.

To respond to the reviewer’s point about the selection of controls, we included 13,648 individuals in longitudinal analysis assessing prediction of incident type 2 diabetes with an integrated risk tool including PRS compared to QDiabetes alone. In preliminary analyses conducted before our first submission, we had used individuals within this sample to optimise parameters for our PRS. In discussion between authors, we felt a clear limitation of this approach was overfitting the constructed PRS to the individuals included in a subsequent analysis using that same PRS. To overcome this, we instead constructed the PRS in the limited number of individuals excluded from the longitudinal analysis (incorrectly described as having “insufficient data”, as the reviewer correctly points out - this has been amended in the text). For many of these individuals, the reason for exclusion was pre-existing type 2 diabetes at study start date (cases), or falling outside the age range for inclusion to the study (25-84) (controls).

Ultimately, the difference in performance of a PRS optimised using these different sets of individuals was negligible (see Fig S1 updated; a marginally different PRS performed best in C+T thresholding, but with a very small difference) and no results were significantly altered in terms of C indices / NRI statistical significance. The effect size of these metrics of performance was marginally attenuated by using the PRS trained on the smaller control group excluded from longitudinal analysis (e.g. net reclassification index for all included participants in Model A was reduced from 3.26% (95% CI 2.04 - 4.41) to 3.22% (95% CI: 2.00–4.38%)). On balance, we feel it is more defensible to present a more conservative estimate of enhancement of risk prediction, and to be confident the PRS does not suffer from overfitting.

5. The authors acknowledge that there may be miscoding of T2D due to misclassification of diabetes as a limitation, but to identify cases, it appears they relied only on the first appearance of the diagnostic code for T2D in the electronic health record. Could they confirm whether this was verified for example, through confirmation of a repeat diagnostic code at a subsequent encounter, prescription data and if not, could they discuss how likely this is to have been an issue in real-world electronic health records.

Both misclassification and miscoding exist within routine electronic health record data, and are not mutually exclusive [[14]](https://paperpile.com/c/8EIZeG/8F0S). In the absence of C-peptide and autoantibody data in our primary care data set, estimating misclassification rates is complex and, in fact, is subject to a separate, ongoing study by our team.

In response to this point, first, we are confident that the ascertainment of diabetes diagnosis in the selected type 2 diabetes population is highly accurate: 98.4% of those included had multiple HbA1c values above a diagnostic threshold for diabetes (HbA1c > 48 mmol/mol).

Second, using prescribing data, we see congruence a typical population of people with type 2 diabetes: 83.1% had received a prescription for either an oral medication used to treat type 2 diabetes, or insulin; with the remaining likely to be receiving lifestyle management alone (as per NICE guidelines [[15]](https://paperpile.com/c/8EIZeG/N4th) in the UK).

Thirdly, we are reassured to find that there are very few people in the type 2 diabetes population we selected who are receiving insulin therapy alone (n=14) which could be more typical of type 1 diabetes.

Unfortunately, the format of primary care data in the Genes & Health 2020 data release does not allow us to see repeated codes, instead researchers to see only the earliest date of diagnosis of any condition; therefore it was not possible to assess for repeat diagnostic codes.

These sanity checks are in keeping with previous reports on the accuracy of electronic health record data, particularly those with conditions such as type 2 diabetes which form part of the Quality Outcome Framework (QOF). QOF forms a major part of how primary care practices are funded in England; previous researchers have reported data miscoding within QOF domains including type 2 diabetes as <2% [[16]](https://paperpile.com/c/8EIZeG/97Vg). While the possibility of miscoding and misclassification is a limitation of this study, on the basis of the above we expect the effect on our results to be small.

We have updated the “limitations” section of our discussion to incorporate these points in brief.

6. What might the effect of a highly consanguineous population be on generation and efficacy of a PRS. The authors say they selected 'unrelated' cases and controls - however, should not a PRS in this specific population take into account high proportions of relatedness? Could the genomic data be used to exclude a given degree of relatedness, measured as "identity by descent" in available software such as PLINK?

PRS in a consanguineous population is expected to be overdispersed (i.e. having higher variance) compared to an outbred population, due to the higher additive variance for non-zero inbreeding coefficient *F* ([[17]](https://paperpile.com/c/8EIZeG/JWYe) page 286). Evaluating whether this is empirically true is somewhat challenging since population structure also affects the variance of a PRS and is correlated with degree of consanguinity in our data. However, we examined this in the largest UMAP-defined cluster in British Pakistanis in G&H, who had higher levels of consanguinity than the British Bangladeshis. We observed that the T2D PRS in individuals in the top 10% of levels of homozygosity in the genome (F_ROH_) showed significantly higher variance than individuals in the bottom 10% (F test p-value 0.038; ratio of variance 1.18). We did not observe significant differences in the accuracy of PRS between subgroups, possibly due to the lack of power (figure below). Additional work is required to further investigate the effects of consanguinity on PRS generation and efficacy, but it is beyond the scope of this paper.

Legend: This plot shows incremental AUC for PRS across clusters in British Bangladeshi and Pakistani individuals in G&H. Grey indicates that the association between PRS and T2D is not significant (p-value >0.05), brown nominally significant, purple significant after bonferroni correction (p-value <0.05/17). Error bars indicate 95% confidence intervals estimated by bootstrapping.

Unfortunately, it is not clear to us what the reviewer means in suggesting that a PRS in this population should take into account high proportions of relatedness. The PRS uses effect size estimates from external cohorts (the multi-ancestry GWAS) so perhaps the reviewer means that *assessment of the PRS accuracy* needs to be undertaken in a set of unrelated individuals. Indeed, we reported the performance of PRS in ‘unrelated’ samples, which is standard in this field. We applied the KING software [[18]](https://paperpile.com/c/8EIZeG/S30X) which used genetic data to estimate the kinship coefficient between individuals in this cohort and we excluded one sample from each pair of 2nd-degree relatives (details now added in the Methods under “Polygenic risk score construction” on page 4). These unrelated individuals (unrelated with each other) still have high levels of consanguinity which is due to them having parents who are related.

The KING software also provides a relatedness estimator based on identity by descent (“PropIBD”). Our previous work investigating the population structure in British Pakistanis by Arciero *et al.* [[19]](https://paperpile.com/c/8EIZeG/Gw4o) showed that this measure gave the highest estimates of kinship between individuals. We performed a sensitivity analysis in a set of unrelated individuals defined using PropIBD. The accuracy of PRS measured by incremental AUC is similar in unrelated individuals defined using this conservative measure (0.031; 95% CI 0.025–0.038; 2658 cases & 9021 controls) compared to what we reported in the paper using a set of unrelated individuals defined using the KING kinship estimator (0.032; 95% CI 0.026–0.039; 2688 cases & 9221 controls). Removing up to 3rd degree relatives showed similar results (0.033; 95% CI 0.026–0.040; 2505 cases & 7923 controls). There is no evidence of inflation using the KING kinship estimator rather than PropIBD to define relatives.

7. There are likely to be a number of reasons why fewer loci were replicated including differences in minor allele frequency, linkage disequilibrium, gene-environment interactions or the simple statistical phenomenon of the "winner's curse" (an overestimate of the effect size reported in the initial study) - a more extensive discussion of this might have been useful.

We thank the reviewer for raising this great point. We accounted for differences in allele frequency and LD in the calculation of power and the proportion of loci that were expected to be transferable (28.1%). After accounting for power, we still observed a lower proportion of replicated loci (22.5%). This is consistent with our observation that the trans-ancestry genetic correlation was significantly lower than 1. As the reviewer pointed out, there might be other reasons such as ancestry-specific gene-environment interactions that we could not account for. In addition, potential overestimation of true genetic effects in the discovery GWAS (the winner’s curse phenomenon) lowers the chances for detecting replication. We have included these points in the second paragraph in discussion on page 9.

8. For the GDM analysis, the sample sizes used to compare PRS between progressors and non-progressors are small. Could the authors verify there was sufficient power based on expected effect sizes from previous studies?

We thank the reviewers for noting this omission. We undertook an additional (retrospective) power analysis, taking an estimate in difference in means of 6.6% from Kwak et al[[20]](https://paperpile.com/c/8EIZeG/0axb). At an alpha level of 0.05, the estimated required total sample size to identify a difference in means is n = 219 (or n ~ 110 per group). Our total sample size was n = 302 (the two groups being compared had sample sizes of n = 175 and n = 127); therefore we expect this aspect of the analysis to be adequately powered. This information has been added to the supplementary methods in Supplementary File 1.

9. Models B and (less so) C that incorporate glycemic measures (glucose and HbA1c, respectively), attenuate the ability of the PRS is predicting T2D onset, suggesting that these biochemical measures probably identify those at risk of T2D. This undermines the utility of the PRS, and confirms previous findings from multiple studies that polygenic prediction of T2D does not add much to clinical predictors if glycemia is included in the instrument (see, e.g. Meigs et al. and Lyssenko et al., both in NEJM 2008). The Lyssenko paper did show that genetic prediction was most useful in younger individuals or those with the longest follow-up, indicating that clinical predictors may not manifest themselves fully until later in life or closer to disease onset, a finding that is consistent with the current study. Since there was no relationship between PRS and incidence of complications, the counter-argument of genetic prediction lacking clinical utility could perhaps be discussed more critically?

Thank you for this helpful suggestion, which we agree merits refinement in our manuscript. We have adjusted the discussion as follows: “The integrated risk tool did not improve NRI compared to model C, which may reflect reduced power in model C due to lower sample size (n=864) than the other two models (n=13,648 for model A; n=4,344 for model B). Alternatively, the lower NRI for models B and C could imply that the benefit of integrating genetic information with risk models already incorporating metrics of hyperglycaemia is more limited than in models without hyperglycaemic metrics (Model A). This interpretation would be in line with previous findings reported in EUR individuals by Lyssenko et al and Meigs et al, although as these studies were performed prior to the availability of any large GWAS data re-assessment with an updated PRS is of benefit, as is its reporting in BPB individuals.”

We also highlight the converse point in discussion - that PRS can be used to stratify individuals, in particular young people, before clinical predictors; that only a single test is required across the life-course; and that risk of multiple conditions can be assessed simultaneously given genotype chip data for an individual [[21]](https://paperpile.com/c/8EIZeG/DOGj).

10. There are multiple statistical comparisons (e.g. all the SNPs taken forward into this cohort), but there were no adjustments to the p-values for multiple comparisons, as a nominal p-value of 0.05 was taken as evidence of replication. While this is sensible when one is trying to estimate how many SNPs will reach that alpha level over the predicted number based on effect size and sample size, this should be acknowledged and explained.

In our experience, multiple testing correction is usually not considered when trying to replicate genetic loci that reached genome-wide significance in previous studies. A nominal significance level (P<0.05) is typically used to assess the evidence of replication.

11. The cluster analysis seems 'tacked on' rather than being integral to the core work of PRS transferability. As such, there is no discussion of the fact that the PRS is associated with different clusters in this analysis but few significant associations with SNPs were found in the original analysis by Ahlqvist et al. The more extensive genetic evaluation undertaken by that group and published in the preprint cited above provides a potential blueprint of what could be done here with the GWAS data obtained in G&H. There should have been some reference to the recent Indian study that undertook clustering and identified a novel insulin-deficient but non-autoimmune cluster (Anjana et al. Pubmed ID 33527813).

Thank you. In response to point 3 from the same reviewer, we have reworked the results and discussion relating to the cluster analysis to ensure that this is an integral part of this work. Specifically, we have developed our argument that the addition of the PRS helps elucidate aetiological subgroups in a real-world setting where measures of insulin secretion/resistance are not routinely available.

We have rectified the omission of this important reference, as well as new references that support the characterisation of a probable SIDD (pSIDD) cluster by its genetic associations [[10,11]](https://paperpile.com/c/8EIZeG/IfOO+4Voh). Furthermore, we have explored the associations of additional polygenic risk scores replicated from the literature with this pSIDD cluster (Figure S5, Table S12, and comments to reviewer 2 above).

12. This is a small point, but the Ahlqvist et al. study preceded and provided the reason for the subsequent Dennis et al. study - since they are cited at the same place in the manuscript, they should be listed in chronological order.

Thank you for noticing this. This has been reordered.

**Reviewer 3**

Comment: The study by Hodgson et al aims at developing a PRS to estimate the 10-year risk of developing T2D and to identify T2D subtypes in patients of South Asian ancestry living in the UK. The authors tested the transferability to South Asians, of genomic variants associated to T2D in people of European descent and then tested the predictive ability of a more South Asian-specific PRS that they combined with the clinical score QDiabetes to estimate the risk of developing T2D in women with gestational diabetes and in men and women, younger or older than 40 years of age. 

Strengths: 
The manuscript is well written, and results are clear.

The research question is important. People of South Asian origin are more affected by T2D. This occurs at a younger age and at lower BMI. The question that there may be a genetically-distinct T2D subtype in this population is sound. Most of PRS have been developed with and for populations of European descent. The transferability of data from Europeans to people of South Asian origin is unknown.

The authors have access to a large set of clinical and genomic data including electronic health records and follow-up of British Pakistani and Bangladeshi (BPB). 

The analyses are appropriate and carefully done. 

The PRS may be useful for women with history of GDM. Women who subsequently developed T2D had a higher PRS than those who did not. Women with GDM had also higher PRS than age- and BMI-matched controls without GDM and higher than men. This clinically important observation could have been investigated further.

We thank the reviewer for these kind comments.

Weaknesses:

1. The construction of the PRS is not detailed enough. The number of SNPs that have been included and their effect size should be stated. How many of the European transferable SNPs have been included in the PRS?

We thank the reviewer for the suggestion. We have added the number of SNPs (2,877) with p-value < 1e-5 and LD r2 <0.1 that were included in the PRS in the Methods. We have included the list of SNPs that were used to construct the PRS in Table S3. The effect sizes of the SNPs can be downloaded from the dbGaP under data accession (phs001672.v4). We cannot share the data publicly due to data access restrictions.

We used all the lead SNPs with p-value < 1e-5 in the PRS, and it included all of the 76 transferable loci which were genome-wide significant (p-value < 5e-8) in the European GWAS, and had p-value <1.1e-6 in the multi-ancestry GWAS.

2. Some of the conclusions are not well supported by the results. For instance, the predictive performance of the PRS over that of the QDiabetes score is very modest. Are the differences in AUC (Table S5) significant after the addition of the PRS? They seem to be significant only for Model A. The incremental effect of the PRS is eliminated by the addition of fasting glucose (Model B) or HbA1c (Model C) to the clinical score.

3. Same comment could be made for the NRI that is significant only for Model A which has the lowest predictive performance. 

We thank the reviewer for highlighting these important points, which are similar to those made by reviewer 2 (comment 9). We have adjusted the discussion as follows: “The integrated risk tool did not improve NRI compared to model C, which may reflect reduced power in model C due to lower sample size (n=864) than the other two models (n=13,648 for model A; n=4,344 for model B). Alternatively, the lower NRI for models B and C could imply that the benefit of integrating genetic information with risk models already incorporating metrics of hyperglycaemia is more limited than in models without hyperglycaemic metrics (Model A). This interpretation would be in line with previous findings reported in EUR individuals by Lyssenko et al and Meigs et al, although as these studies were performed prior to the availability of any large GWAS data re-assessment with an updated PRS is of benefit, as is its reporting in BPB individuals. We have adjusted findings throughout the discussion to refer to a “modest” improvement in predictive performance, and emphasised this is only relevant to Model A.”

We also highlight the converse point in discussion - that PRS can be used to stratify individuals, in particular young people, before clinical predictors; that only a single test is required across the life-course; and that risk of multiple conditions can be assessed simultaneously.

4. Data-driven clustering: T2D cluster driven by the PRS is the largest in term of number of individuals. However, the progression of microvascular and macrovascular outcomes in individuals of this cluster does not differ from the other clusters. The only cluster that differs between macro and microvascular complications is the MARD one. This could be explained by the fact that age has a different impact on the progression of macrovascular and microvascular outcomes. 
The cluster analyses could be done also individuals of European descent and the radar plots compared between the two ethnic groups. 

Individuals in the MARD cluster progressed to both micro- and macro-vascular complications at a faster rate than other clusters. We did not control for age in the presented survival analyses, after lengthy discussions within the analysis team, as age was one of the key variables used to derive clusters - we would expect controlling for age in the survival analysis to undermine any effect of age in delineated clusters.

As expected, in age-adjusted survival analyses the rate of progression to complications was not different in the MARD group, suggesting, as the reviewer highlights, that it is age driving this difference in rates of progression in this group. These results are included in the results section of the manuscript.

The reviewer makes an excellent point that it would be interesting to compare clustering between people of European descent and British Pakistani and Bangladeshi individuals. Performing a substantial cluster analysis on a separate data set of EUR individuals would be a substantial analytic undertaking beyond the scope of this paper. Furthermore, it may add relatively little to the literature in view of the recent publication by Mansour Aly et al. [[11]](https://paperpile.com/c/8EIZeG/4Voh) describing genetic differences between clusters in people of European descent previously defined by the Ahlqvist group. Comparison with these studies was not the primary focus of this work, but could be the subject of further study in the future.

Minor:

The title should be more specific to the findings.

We thank the reviewer for the suggestion. The title has been changed to “Integrating polygenic risk scores in prediction of type 2 diabetes risk and subtypes: an analysis of genomic and routine primary care health data in a population-based study of British Pakistanis and Bangladeshis”.

**References**

1.	[Finer S, Martin HC, Khan A, Hunt KA, MacLaughlin B, Ahmed Z, et al. Cohort Profile: East London Genes & Health (ELGH), a community-based population genomics and health study in British Bangladeshi and British Pakistani people. Int J Epidemiol. 2020;49: 20–21i.](http://paperpile.com/b/8EIZeG/Fu6P)

2.	[Robson J, Dostal I, Madurasinghe V, Sheikh A, Hull S, Boomla K, et al. NHS Health Check comorbidity and management: an observational matched study in primary care. British Journal of General Practice. 2017. pp. e86–e93. doi:](http://paperpile.com/b/8EIZeG/KL28)[10.3399/bjgp16x688837](http://dx.doi.org/10.3399/bjgp16x688837)

3.	[Khera AV, Chaffin M, Aragam KG, Haas ME, Roselli C, Choi SH, et al. Genome-wide polygenic scores for common diseases identify individuals with risk equivalent to monogenic mutations. Nat Genet. 2018;50: 1219–1224.](http://paperpile.com/b/8EIZeG/geV2)

4.	[Khera AV, Chaffin M, Wade KH, Zahid S, Brancale J, Xia R, et al. Polygenic Prediction of Weight and Obesity Trajectories from Birth to Adulthood. Cell. 2019;177: 587–596.e9.](http://paperpile.com/b/8EIZeG/3H3o)

5.	[Giambartolomei C, Vukcevic D, Schadt EE, Franke L, Hingorani AD, Wallace C, et al. Bayesian test for colocalisation between pairs of genetic association studies using summary statistics. PLoS Genet. 2014;10: e1004383.](http://paperpile.com/b/8EIZeG/KQLj)

6.	[Colocalisation Tests of Two Genetic Traits [R package coloc version 5.1.0]. 2021 [cited 13 Dec 2021]. Available:](http://paperpile.com/b/8EIZeG/pIyt) [https://CRAN.R-project.org/package=coloc](https://cran.r-project.org/package=coloc)

7.	[Kooner JS, Saleheen D, Sim X, Sehmi J, Zhang W, Frossard P, et al. Genome-wide association study in individuals of South Asian ancestry identifies six new type 2 diabetes susceptibility loci. Nat Genet. 2011;43. doi:](http://paperpile.com/b/8EIZeG/Z1vh)[10.1038/ng.921](http://dx.doi.org/10.1038/ng.921)

8.	[Vujkovic M, Keaton JM, Lynch JA, Miller DR, Zhou J, Tcheandjieu C, et al. Discovery of 318 new risk loci for type 2 diabetes and related vascular outcomes among 1.4 million participants in a multi-ancestry meta-analysis. Nat Genet. 2020;52: 680–691.](http://paperpile.com/b/8EIZeG/7M0H)

9.	[Márquez-Luna C, Loh P-R, South Asian Type 2 Diabetes (SAT2D) Consortium, SIGMA Type 2 Diabetes Consortium, Price AL. Multiethnic polygenic risk scores improve risk prediction in diverse populations. Genet Epidemiol. 2017;41: 811–823.](http://paperpile.com/b/8EIZeG/By10)

10.	[Prasad RB, Asplund O, Shukla SR, Wagh R, Kunte P, Bhat D, et al. Subgroups of patients with young-onset type 2 diabetes in India reveal insulin deficiency as a major driver. Diabetologia. 2022;65: 65–78.](http://paperpile.com/b/8EIZeG/IfOO)

11.	[Mansour Aly D, Dwivedi OP, Prasad RB, Käräjämäki A, Hjort R, Thangam M, et al. Genome-wide association analyses highlight etiological differences underlying newly defined subtypes of diabetes. Nat Genet. 2021;53: 1534–1542.](http://paperpile.com/b/8EIZeG/4Voh)

12.	[Udler MS, Kim J, von Grotthuss M, Bonàs-Guarch S, Cole JB, Chiou J, et al. Type 2 diabetes genetic loci informed by multi-trait associations point to disease mechanisms and subtypes: A soft clustering analysis. PLoS Med. 2018;15: e1002654.](http://paperpile.com/b/8EIZeG/qYlQ)

13.	[Ahlqvist E, Storm P, Käräjämäki A, Martinell M, Dorkhan M, Carlsson A, et al. Novel subgroups of adult-onset diabetes and their association with outcomes: a data-driven cluster analysis of six variables. The lancet Diabetes & endocrinology. 2018;6. doi:](http://paperpile.com/b/8EIZeG/7yHA)[10.1016/S2213-8587(18)30051-2](http://dx.doi.org/10.1016/S2213-8587(18)30051-2)

14.	[de Lusignan S, Sadek N, Mulnier H, Tahir A, Russell-Jones D, Khunti K. Miscoding, misclassification and misdiagnosis of diabetes in primary care. Diabet Med. 2012;29. doi:](http://paperpile.com/b/8EIZeG/8F0S)[10.1111/j.1464-5491.2011.03419.x](http://dx.doi.org/10.1111/j.1464-5491.2011.03419.x)

15.	[Overview | Type 2 diabetes in adults: management | Guidance | NICE. [cited 13 Dec 2021]. Available:](http://paperpile.com/b/8EIZeG/N4th) <https://www.nice.org.uk/guidance/ng28>

16.	[Rowena Hilson CG. Coding, Classification and Diagnosis of Diabetes. RCGP. Available:](http://paperpile.com/b/8EIZeG/97Vg) [(https://orchid.phc.ox.ac.uk/wp-content/uploads/2017/02/nhs_diabetes_and_rcgp_cod_final_report.pdf)](about:blank)

17.	[Frankham R. Introduction to quantitative genetics (4th edn). Trends in Genetics. 1996. p. 280. doi:](http://paperpile.com/b/8EIZeG/JWYe)[10.1016/0168-9525(96)81458-2](http://dx.doi.org/10.1016/0168-9525(96)81458-2)

18.	[Manichaikul A, Mychaleckyj JC, Rich SS, Daly K, Sale M, Chen W-M. Robust relationship inference in genome-wide association studies. Bioinformatics. 2010;26: 2867–2873.](http://paperpile.com/b/8EIZeG/S30X)

19.	[Arciero E, Dogra SA, Malawsky DS, Mezzavilla M, Tsismentzoglou T, Huang QQ, et al. Fine-scale population structure and demographic history of British Pakistanis. Nat Commun. 2021;12: 1–16.](http://paperpile.com/b/8EIZeG/Gw4o)

20.	[Kwak SH, Choi SH, Kim K, Jung HS, Cho YM, Lim S, et al. Prediction of type 2 diabetes in women with a history of gestational diabetes using a genetic risk score. Diabetologia. 2013;56: 2556–2563.](http://paperpile.com/b/8EIZeG/0axb)

21.	[Lambert SA, Abraham G, Inouye M. Towards clinical utility of polygenic risk scores. Hum Mol Genet. 2019;28: R133–R142.](http://paperpile.com/b/8EIZeG/DOGj)
