# Supplementary material for: Integrating polygenic risk scores in the prediction of type 2 diabetes risk and subtypes in British Pakistanis and Bangladeshis: A population-based cohort study
Source: PLoS Med. 2022 May 19;19(5):e1003981. doi: 10.1371/journal.pmed.1003981 (PMC9119501; doi:10.1371/journal.pmed.1003981)
Supplement: Attachment — Submitted filename: Manuscript Number PMEDICINE.docx [file pmed.1003981.s023.docx]

**Manuscript Number PMEDICINE-D-21-03668
"Integrating polygenic risk scores in the prediction of type 2 diabetes risk and subtypes: an analysis of genomic and routine primary care health data in a population-based study of British Pakistanis and Bangladeshis"**

The modifications made to the revised version of the manuscript clearly add to the quality of this paper, and most of my concerns from my previous review have been addressed. I recommend an accept of the manuscript.
