# Supplementary material for: Integrating polygenic risk scores in the prediction of type 2 diabetes risk and subtypes in British Pakistanis and Bangladeshis: A population-based cohort study
Source: PLoS Med. 2022 May 19;19(5):e1003981. doi: 10.1371/journal.pmed.1003981 (PMC9119501; doi:10.1371/journal.pmed.1003981)
Supplement: Attachment — Submitted filename: PLoS_Medicine_Reviewers_Comments_2022Feb.docx [file pmed.1003981.s024.docx]

**Requests from the editors:**

The current title is slightly too long. Please update to 'Integrating polygenic risk scores in the prediction of type 2 diabetes risk and subtypes in British Pakistanis and Bangladeshis: a population-based cohort study', or similar.

Thank you. We have shortened the title and changed it to your suggested wording.

Thanks for providing a completed TRIPOD checklist - please update the checklist to use section and paragraph numbers as opposed to page numbers (as these change during the revision process).

Please also ensure the TRIPOD checklist is cited in the methods (e.g. 'This study is reported according to the Transparent reporting of a multivariable prediction model for individual prognosis or diagnosis (TRIPOD) statement (S1 checklist)'.

These changes have been made.

Comments from the reviewers:

**Reviewer #1**: The modifications made to the revised version of the manuscript clearly add to the quality of this paper, and most of my concerns from my previous review have been addressed. I recommend an accept of the manuscript.

Thank you very much for this favourable assessment of our revisions and manuscript.

**Reviewer #2**: The authors have been very responsive to the review process and the manuscript is significantly improved. Particularly I appreciate the new designation of the pSIDD cluster. There is a small number of minor outstanding issues that should be relatively straightforward to resolve.

We appreciate this comment, thank you, and will address the minor outstanding issues as below:

1.	The choice of SNPs to construct the most informative polygenic score: thank you for the clarification, and it does make sense to use the multiethnic resource that includes more samples than Kooner et al. I still think that some mention of the rationale used and elegantly outlined in the response to the reviewers should find its way into the manuscript, if anything to allay the concern of the informed reader. This could be done in the Supplement or as a couple of sentences in the Methods.

We thank the reviewer for the suggestion and agree with the need to include further mention of the approach we took. We have now added the rationale in the **Methods** section (the last paragraph on page 4) and detailed methods and results in the **Supplementary File** **1** (section “PRS using a South Asian-specific GWAS“ on page 2–3).

2.	Multiple testing correction for SNPs taken forward: I agree that for a replication of a single variant, a P-value <0.05 is sufficient. When one is testing replication of multiple individual variants, the number of hypotheses is as large as the number of uncorrelated variants taken forward: thus strictly speaking it does make sense to use a Bonferroni correction, and this is done routinely. You could simply state that the variants passed the nominal P-value, but that experiment-wide significance would be set at 0.05/n.

Thank you for this comment and further clarification of it. We have now added the number of loci (n=13) that replicated at p-value = 1.5✕10^-4^ (0.05/338; Bonferroni correction) in G&H in **Results** (page 7), **Table S6**, **S7** (Column “Significant_Bonferroni” & “power at alpha=0.05/338”) and **Supplementary File 1** (page 2). We have also estimated that at the alpha = 0.05/338 level, we expect that 3.1% of the loci (n=11) are powered to replicate in G&H, which is not significantly different from the observed proportion of transferred loci (3.8%; **Supplementary File 1**). Given that the discovery GWAS was done in more than one million samples, the power to replicate at this more stringent significance level is extremely low in our current study, and larger South Asian cohorts are needed for replication at this significance level.

3.	As minor edits, in Supplementary Table 4, the locus "CDKAL1" has a typo ("CKDAL1"). Gene names should be italicized throughout, including Supplementary Tables

Thank you for spotting this mistake which we have now fixed in **Table S4** and updated gene names in **Table S6**, **S7**.

**Reviewer #3**: The modifications made to the revised version of the manuscript clearly add to the quality of this paper, and most of my concerns from my previous review have been addressed adequately.

Thank you very much for this positive assessment.

**Reviewer #4**: The authors explore the genetic determinants of Type 2 Diabetes (T2D) in people of British Pakistani and Bangladeshi (BPB) ancestry. Using genetic data from the Genes & Health project, they first explore whether SNPs associated with T2D in European populations are also associated with T2D in their BPB sample. They then construct a polygenic risk score (PRS) for T2D and show that it can be useful for predicting which individuals will develop T2D alongside an already established clinical tool (QDiabetes).

I would like to commend the authors for working with a previously understudied population group, and I agree with them that further genetic research involving such groups is needed.

We thank the reviewer for the comment.

As a statistician, I am perhaps unable to fully scrutinise the authors' applied results, but I will attempt to provide some comments on their use of appropriate methodology to obtain these results. Since I was not involved in the previous round of review, some of my comments will be complementary to those previously raised by other reviewers, while other comments will be different.

Here are my comments on the manuscript.

1)	The authors used a European LD panel (European 1000G samples) for their LD clustering and thresholding analysis. Was it not possible to use a south Asian panel instead?

We used GWAS summary data derived from a large-scale multi-ancestry meta-analysis by Vujkovic *et al*. [[1]](https://paperpile.com/c/6u8FrQ/8llR), as this was the best way to boost predictive power and therefore offered significant advantages over existing south Asian GWAS (as was discussed at length in the previous response to reviewer 2). When constructing polygenic scores from GWAS summary statistics using the clumping and thresholding method, we should always use a reference panel that matches the ancestry of the GWAS samples. Ideally, we should use an LD reference panel comprising individuals from the original GWAS. However, since the individual-level data are usually unavailable, a reference sample (from e.g. 1000 Genomes Project) of the same ancestry should be used to estimate the LD correlation (see the second paragraph of the “Controlling for LD” section in this tutorial paper [[2]](https://paperpile.com/c/6u8FrQ/wiUj)). A large proportion (79%) of the GWAS samples in the Vujkovic *et al*. study had European ancestry, so we used 1000 Genomes European samples to approximate the LD values, which has been shown to work well in other studies that used multi-ancestry GWAS data [[3–5]](https://paperpile.com/c/6u8FrQ/JY2e+SKDI+Ok3L).

2)	In their supplement, the authors present a formula according to which they calculated the proportion of SNPs from the European GWAS that would be expected to replicate in the G+H GWAS. First of all, the formula seems wrong; for example, in a case-control study with n_cases = n_controls, one would have 1 - phi = 0 and hence only a 0.05 probability to replicate a SNP regardless of its beta coefficient in the European GWAS. Please clarify (could it be that phi = n_cases / n instead?). Moreover, the authors should provide an explanation about why the formula does what they are trying to achieve, or a reference to support its use. In general, I found it a bit surprising that 72% of these GWAS-significant variants should be expected to not associate with T2D even at a nominal 0.05 level in the authors' genetic sample.

We thank the reviewer for pointing out the error. The reviewer was correct and phi should be n_cases / n instead. We have now fixed this error and calculated power. The proportion of loci that were expected to replicate in G&H is now 30% (previously 28%). Our conclusion is unchanged, i.e. that we observed fewer transferable loci than would be expected. The **Abstract,** **Results** on page 7, and **Table S6** have now been updated.

We have now added the references [[6,7]](https://paperpile.com/c/6u8FrQ/4VwU0+1JgEJ) to **Supplementary File 1** (page 1, reference 8 and 9). We modified the R codes by Matti Pirinen [[7]](https://paperpile.com/c/6u8FrQ/1JgEJ). These equations are routinely used to estimate the power, and empirical findings tend to match the expectations well (e.g. [[8,9]](https://paperpile.com/c/6u8FrQ/woBY+cELf)).

It may seem surprising that we do not have power to replicate 70% of the genome-wide significant loci at a nominal 0.05 level in G&H, but we would like to note that the discovery European GWAS was conducted in more than 1.1 million samples and is much more powerful than G&H, which has only ~20,000 samples. It is therefore expected that attempts to replicate associations that are identified in large European-ancestry samples in much smaller non-European cohorts will be underpowered since the effect sizes of most common genetic variants are so small.

3)	In the authors' colocalization analyses, the overlap of genetic variants between the European and G+H GWAS in each region was as low as 10%. Therefore, it is quite likely that in some regions the truly causal variant(s) may not have been genotyped in one of the two samples (or even in none of the two samples). Would that be a problem for the TEColoc method used by the authors?

We thank the reviewer for this excellent question. TEColoc is based on the Joint likelihood mapping (JLIM) model developed by Chun *et al.* [[10]](https://paperpile.com/c/6u8FrQ/9qUK). This method tests whether the association signals in two datasets can be explained by the same underlying causal variant, without identifying the specific causal variant (as described in **Supplementary File 1**, page 2), which is an advantage of the colocalisation method. It is not a problem (and indeed not uncommon) that the causal variant(s) are not genotyped or well imputed in any or neither of the datasets. We totally agree with the reviewer and there is definitely a lower limit of coverage that is acceptable. We need a sufficient number of tagging variants (i.e. those are correlated with the true causal variants) in both datasets to detect a significant colocalisation signal. The method does not perform well with very low coverage and the threshold (10%) was determined empirically. For loci that did not show significant evidence of colocalisation, we could not rule out the possibility of shared causal variants that we failed to detect because of the low coverage (false negatives), in addition to other reasons such as low statistical power in Genes & Health or multiple causal variants that lead to complex association signals and LD patterns. However, low coverage is not likely to cause false positives and we are confident in the significant colocalisation signals that we identified. We have added the following sentence to the **Methods** section on page 4: “We might not be able to detect shared causal variants due to the low coverage for some loci, but it is not likely to cause false positives.”

4)	What was the explanatory power (e.g. in terms of R^2 values) of the diabetes PRS constructed by the authors? Also, did the PRS associate with any of the other variables included in their analysis (BMI, HDL, TG, HBA1c, glucose)? Following from a previous comment by another reviewer, any associations with glucose and HBA1c would be particularly interesting to report, since they would explain why the PRS offered little benefit over QDiabetes when using models B or C which incorporated these traits.

We thank the reviewer for the suggestion. We have now calculated the pseudo R^2^ on the liability scale [[11]](https://paperpile.com/c/6u8FrQ/Goqe). We used 13.6% as the prevalence estimate for T2D in South Asian-ancestry individuals in the UK, the background population from which Genes & Health is sampled, defined as all people from South Asian ethnicities (n=255,066 aged ≥20 years) registered with a primary health physician/GP in four east London boroughs. The additional R^2^ on the liability scale of our T2D PRS on top of covariates is 4.6% (95% confidence interval: 3.7%–5.6%). We have now added this result in the manuscript (page 5 & 7).

We explored associations between PRS and the variables the reviewer suggested, and have added a new table to the manuscript (**Table S8**) and updated the text (Page 7, para 3) to describe these. We described the association between PRS and the variables at (1) the longitudinal incident disease prediction start date (ie, May 2010, for all 13648 individuals without pre-existing type 2 diabetes aged 25-84 years), and (2) the time of diagnosis, for individuals with type 2 diabetes. We observed similar results in both settings. As the reviewer suggested may be the case, there is a statistically significant association between PRS and both HbA1c and fasting glucose. However, the strength of the association is weak (Pearson coefficients 0.11 (FPG) and 0.07 (HbA1c) at the study start date, and 0.08 (FPG) and 0.12 (HbA1c) at the time of type 2 diabetes diagnosis). This association could partly explain our existing observation that the PRS does not enhance risk prediction on top of QDiabetes models B and C compared to model A - this perspective has been added to the discussion (page 10, para 3).

5)	To assess the predictive accuracy of their PRS, the authors use the net reclassification index (NRI), which amounts to first binarizing each method's predictions by splitting individuals to high-risk and low-risk groups and then comparing the proportions of cases in each group. Wouldn't it make more sense to not binarize and instead compare each method's predictive accuracy using the actual estimated probabilities? E.g. something like Sum( |Pr(case) - Case.status| ) where Case.status = 1 for cases and 0 for controls, and the summation extends over all individuals.

We thank the reviewer for the comment. It is true that this approach could summarise and reflect absolute risk estimates and the magnitude of the differences between the IRT and QDiabetes risk predictions. We have calculated this suggested metric of predictive accuracy as the reviewer suggests, with values in **Table 1** below this paragraph. According to this metric, a score of 0 would be perfect model performance, and a score of n would be absolutely imperfect model performance (where n is the number of individuals assessed). Because the summation is a function of the number of individuals assessed, to allow comparison between different QDiabetes models we felt it more appropriate to consider the mean (ie (Sum (|Pr(case) - Case.status| ) / n), where Case.status = 1 for cases and 0 for non-cases. By dividing by n the metric then fits a scale of 0-1, with 0 being perfect model performance, and 1 being worst possible performance.

**Table 1: Relative performance of QDiabetes and Integrated Risk Tool (IRT) models for prediction of incident type 2 diabetes, calculated using the formula suggested by the reviewer: Sum( |Pr(case) - Case.status| ) where Case.status = 1 for cases and 0 for controls. Data are presented for QDiabetes models A, B, and C (QDA, QDB, QDC) as described in the manuscript.**

|  | QDA | QDA IRT | QDB | QDB IRT | QDC | QDC IRT |
| --- | --- | --- | --- | --- | --- | --- |
| n | 13648 | 13648 | 4334 | 4334 | 864 | 864 |
| Sum(\|Pr(case) - case.status\|) | 3439.3 | 3398.4 | 1170.2 | 1157.2 | 291.2 | 286.8 |
| Mean(\|Pr(case) - case.status\|) | 0.252 | 0.249 | 0.270 | 0.267 | 0.337 | 0.332 |
| 95% Confidence interval (lower) | 0.247 | 0.244 | 0.260 | 0.257 | 0.316 | 0.312 |
| 95% Confidence interval (upper) | 0.257 | 0.254 | 0.280 | 0.277 | 0.358 | 0.352 |

However, this metric suggested by the reviewer is not commonly used in existing literature on this topic (to our knowledge) so it is not possible to compare this to other studies. Furthermore, we think that the binary approach (i.e. the categorical NRI) has more clinical relevance, because type 2 diabetes risk estimates do lead to a ‘binarised’ decision at to whether a patient will be offered a clinical intervention or not. The NRI estimates are commonly used in assessing the clinical utility of PRS, e.g. [[12–15]](https://paperpile.com/c/6u8FrQ/Fk70+73TZ+rQyh+6iS7). In line with the reviewer’s suggestion for a non-binarized metric, we have now also calculated the continuous NRI (Table S9), which focuses on whether cases or controls had higher or lower risk estimates by the integrated score than QDiabetes alone, respectively (page 6, para 1). The continuous NRI does not rely on an arbitrary cutoff that determines the high-risk and low-risk groups, but does tend to give a more generous estimate of model enhancement. The categorical NRI tends to be presented for PRS-enhanced risk prediction models [[12–15]](https://paperpile.com/c/6u8FrQ/Fk70+73TZ+rQyh+6iS7). Therefore, we elected to present the more conservative and clinically-relevant estimates provided by categorical NRI throughout the manuscript.

6)	One possible limitation of the study is the potential for its results to be affected by selection bias. For example, since BPB individuals were only recruited at three centres across the UK (East London, Bradford and Manchester), some of their baseline characteristics may differ to those of other such populations. The construction of the PRS was based on an even more selective sample, since individuals included in the QDiabetes assessment sample were excluded from the PRS construction. In addition, genetic studies conducted on individuals with a pre-established disease are known to suffer from a form of selection bias called index event bias; this may have been present in the authors' analysis on T2D incidence in women who had previously developed gestational diabetes.

The reviewer is right that the BPB individuals used in our study are not a random sample of all BPB individuals in the UK. Indeed, there are very few large cohort studies of this kind that do not have some kind of selection bias due to how recruitment is carried out. The key question is whether this is likely to have affected our conclusions.

The power of the discovery GWAS is the main factor that determines the performance of the PRS. Our T2D PRS was derived from an independent, large-scale muti-ancestry GWAS conducted in 1.4 million samples by Vujkovic *et al*. [[1]](https://paperpile.com/c/6u8FrQ/8llR) (page 4-5). We used the samples that were not included in the longitudinal analysis to select the best parameter to avoid overfitting, but the performance of the best C+T score in the longitudinal analysis was actually similar to that selected in the tuning set, as shown in **Figure S1A**. This suggests that using a selected sample has not greatly affected our inference here.

In the GDM analysis, we observed that in women who had a history of GDM, those who subsequently developed T2D had higher PRS than those who did not. The same is also observed in women without a history of GDM, and in all women regardless of the history of GDM, which suggests that the impact of index event bias (or collider bias) is minimal, otherwise we would have observed distorted association in women with GDM which would be different from the association in all women (i.e. we would be more prone to the index event bias if we claimed a very different association in the subgroup).

7)	As mentioned by the authors, one of the main reasons for studying T2D in BPB individuals is the increased frequency of diabetes in this population group. Can any of the authors' findings help to explain why T2D is more common in BPB individuals?

We thank the reviewer for the comment. We agree that it is an interesting and important question, but unfortunately it is beyond the scope of the paper. As we do not have a non-BPB cohort to compare to (and selected in the same way), it is not possible for us to attempt to explain whether polygenic risk plays an important role in the higher prevalence of T2D in BPB individuals. Furthermore, there are major challenges to studying this due to the complex confounding influences of genetics, behavioural, socioeconomic and cultural factors.

8)	In their conclusions, the authors state that the addition of their PRS to data-driven identification of T2D subgroups can improve the classification of individuals into T2D subgroups. It is perhaps worth clarifying that this seems to be the case only if information on other relevant variables is missing. In general, the results of a statistical clustering algorithm depend on what variables the clustering was based on. Presumably, the authors' PRS was only needed to identify and characterize the pSIDD cluster because information on fasting glucose and beta cell function-related traits was not available in their dataset, and the PRS would be redundant if such information was available.

We thank the reviewer for this excellent point. While information on these other relevant variables could clearly improve clustering, these are very challenging to measure and interpret correctly and therefore are rarely measured in clinical practice in the management of type 2 diabetes, particularly in primary care, where the majority of patients are cared for. The reason these metrics were absent from our data set is precisely for this reason: because our data set reflects real world management of real world patients. These tests are expensive, complex to measure, and challenging to interpret properly, and it is unlikely they will be integrated into routine community care of type 2 diabetes. In this context, a PRS has the potential to be a much more pragmatic tool as it can be calculated from a genotyping chip which generates stable estimates of genetic risk across multiple traits and which could be performed from a saliva sample at any age. We have updated the discussion (**page 11)** to address this point.

**References**

1.	[Vujkovic M, Keaton JM, Lynch JA, Miller DR, Zhou J, Tcheandjieu C, et al. Discovery of 318 new risk loci for type 2 diabetes and related vascular outcomes among 1.4 million participants in a multi-ancestry meta-analysis. Nat Genet. 2020;52: 680–691.](http://paperpile.com/b/6u8FrQ/8llR)

2.	[Choi SW, Mak TS-H, O’Reilly PF. Tutorial: a guide to performing polygenic risk score analyses. Nat Protoc. 2020;15: 2759–2772.](http://paperpile.com/b/6u8FrQ/wiUj)

3.	[Wang Y, Namba S, Lopera E, Kerminen S, Tsuo K, Läll K, et al. Global biobank analyses provide lessons for computing polygenic risk scores across diverse cohorts. bioRxiv. 2021. doi:](http://paperpile.com/b/6u8FrQ/JY2e)[10.1101/2021.11.18.21266545](http://dx.doi.org/10.1101/2021.11.18.21266545)

4.	[Wang M, Menon R, Mishra S, Patel AP, Chaffin M, Tanneeru D, et al. Validation of a Genome-Wide Polygenic Score for Coronary Artery Disease in South Asians. J Am Coll Cardiol. 2020;76: 703–714.](http://paperpile.com/b/6u8FrQ/SKDI)

5.	[Khera AV, Chaffin M, Aragam KG, Haas ME, Roselli C, Choi SH, et al. Genome-wide polygenic scores for common diseases identify individuals with risk equivalent to monogenic mutations. Nat Genet. 2018;50: 1219–1224.](http://paperpile.com/b/6u8FrQ/Ok3L)

6.	[Sham PC, Purcell SM. Statistical power and significance testing in large-scale genetic studies. Nat Rev Genet. 2014;15: 335–346.](http://paperpile.com/b/6u8FrQ/4VwU0)

7.	[Matti Pirinen, University of Helsinki. GWAS 3: Statistical power. [cited 10 Feb 2022]. Available:](http://paperpile.com/b/6u8FrQ/1JgEJ) <https://www.mv.helsinki.fi/home/mjxpirin/GWAS_course/material/GWAS3.html>

8.	[Giannakopoulou O, Lin K, Meng X, Su M-H, Kuo P-H, Peterson RE, et al. The Genetic Architecture of Depression in Individuals of East Asian Ancestry: A Genome-Wide Association Study. JAMA Psychiatry. 2021;78: 1258–1269.](http://paperpile.com/b/6u8FrQ/woBY)

9.	[Huang QQ, Sallah N, Dunca D, Trivedi B, Hunt KA, Hodgson S, et al. Transferability of genetic loci and polygenic scores for cardiometabolic traits in British Pakistanis and Bangladeshis. bioRxiv. medRxiv; 2021. doi:](http://paperpile.com/b/6u8FrQ/cELf)[10.1101/2021.06.22.21259323](http://dx.doi.org/10.1101/2021.06.22.21259323)

10.	[Chun S, Casparino A, Patsopoulos NA, Croteau-Chonka DC, Raby BA, De Jager PL, et al. Limited statistical evidence for shared genetic effects of eQTLs and autoimmune-disease-associated loci in three major immune-cell types. Nat Genet. 2017;49: 600–605.](http://paperpile.com/b/6u8FrQ/9qUK)

11.	[Lee SH, Goddard ME, Wray NR, Visscher PM. A better coefficient of determination for genetic profile analysis. Genet Epidemiol. 2012;36: 214–224.](http://paperpile.com/b/6u8FrQ/Goqe)

12.	[Sun L, Pennells L, Kaptoge S, Nelson CP, Ritchie SC, Abraham G, et al. Polygenic risk scores in cardiovascular risk prediction: A cohort study and modelling analyses. PLOS Medicine. 2021. p. e1003498. doi:](http://paperpile.com/b/6u8FrQ/Fk70)[10.1371/journal.pmed.1003498](http://dx.doi.org/10.1371/journal.pmed.1003498)

13.	[Mars N, Koskela JT, Ripatti P, Kiiskinen TTJ, Havulinna AS, Lindbohm JV, et al. Polygenic and clinical risk scores and their impact on age at onset and prediction of cardiometabolic diseases and common cancers. Nat Med. 2020;26: 549–557.](http://paperpile.com/b/6u8FrQ/73TZ)

14.	[Riveros-Mckay F, Weale ME, Moore R, Selzam S, Krapohl E, Sivley RM, et al. An Integrated Polygenic Tool Substantially Enhances Coronary Artery Disease Prediction. Circ Genom Precis Med. 2021. doi:](http://paperpile.com/b/6u8FrQ/rQyh)[10.1161/CIRCGEN.120.003304](http://dx.doi.org/10.1161/CIRCGEN.120.003304)

15.	[Mosley JD, Gupta DK, Tan J, Yao J, Wells QS, Shaffer CM, et al. Predictive Accuracy of a Polygenic Risk Score Compared With a Clinical Risk Score for Incident Coronary Heart Disease. JAMA. 2020;323: 627–635.](http://paperpile.com/b/6u8FrQ/6iS7)
